# Genome-Wide Chromatin Landscape Transitions Identify Novel Pathways in Early Commitment to Osteoblast Differentiation

**DOI:** 10.1371/journal.pone.0148619

**Published:** 2016-02-18

**Authors:** Bethtrice Thompson, Lyuba Varticovski, Songjoon Baek, Gordon L. Hager

**Affiliations:** 1 Laboratory of Receptor Biology and Gene Expression, NCI, NIH, Bethesda, MD, United States of America; 2 Center for Cancer Research and Therapeutic Development, Clark Atlanta University, Atlanta, GA, United States of America; University of Massachusetts Medical, UNITED STATES

## Abstract

Bone continuously undergoes remodeling by a tightly regulated process that involves osteoblast differentiation from Mesenchymal Stem Cells (MSC). However, commitment of MSC to osteoblastic lineage is a poorly understood process. Chromatin organization functions as a molecular gatekeeper of DNA functions. Detection of sites that are hypersensitive to Dnase I has been used for detailed examination of changes in response to hormones and differentiation cues. To investigate the early steps in commitment of MSC to osteoblasts, we used a model human temperature-sensitive cell line, hFOB. When shifted to non-permissive temperature, these cells undergo "spontaneous" differentiation that takes several weeks, a process that is greatly accelerated by osteogenic induction media. We performed Dnase I hypersensitivity assays combined with deep sequencing to identify genome-wide potential regulatory events in cells undergoing early steps of commitment to osteoblasts. Massive reorganization of chromatin occurred within hours of differentiation. Whereas ~30% of unique DHS sites were located in the promoters, the majority was outside of the promoters, designated as enhancers. Many of them were at novel genomic sites and need to be confirmed experimentally. We developed a novel method for identification of cellular networks based solely on DHS enhancers signature correlated to gene expression. The analysis of enhancers that were unique to differentiating cells led to identification of bone developmental program encompassing 147 genes that directly or indirectly participate in osteogenesis. Identification of these pathways provided an unprecedented view of genomic regulation during early steps of differentiation and changes related to WNT, AP-1 and other pathways may have therapeutic implications.

## Introduction

MSC were first recognized in the bone marrow by a German pathologist, Julius Cohnheim in 1867 by the presence of non-hematopoietic cells with a fibroblast-like morphology [1]. Fifty years later Alexander Friedenstein characterized these cells as colony-forming unit fibroblasts, and demonstrated that these cells can differentiate into bone-forming cells, later named Mesenchymal stem cells (MSC) and osteoblasts (OB) [2], The three stages of bone formation: proliferation, matrix maturation, and mineralization have been traditionally defined by sequential expression of cell growth and differentiation-related transcription factors (TFs) [2], [3] such as Runx2 [4], [5], [6], Osterix/Sp7, and others [7], [8]. More recently, MSC were also found to have the potential to differentiate into chondroblasts, adipocytes and myoblasts [8], [9] and these processes are also characterized by time-wise expression of specific TFs and other genes. Previous studies of MSC differentiation used available tools such as RNA expression by microarray analysis [10], [11] and chromatin immunoprecipitation (ChIP) for specific DNA-binding proteins [2] [12]. However, these studies have not provided broad information on all regulatory elements involved in MSC differentiation.

Chromatin organization functions as a molecular gatekeeper of cellular function permitting accessibility of TFs to precise DNA sites. ATP-dependent chromatin remodeling complexes, DNA methylases and histone modifying enzymes lead to specific modifications in chromatin structure that allow communication between transcriptional machinery and DNA. The sites on DNA that interact with transcription factors contain disorganized nucleosome structures, and are thus hypersensitive to DNA nucleases. Detection of sites hypersensitive to Dnase I, followed by isolation and deep sequencing of fragments (DHS-seq) has recently been adapted to obtain functional analysis of the entire accessible genome (i. e. all sites in the genome potentially accessible to transcriptional machinery at any time) [13], [14]. In contrast to ChIP-seq, which provides a targeted view that is limited to known DNA-binding proteins and covers only a fraction of sites [12,15–17], DHS-seq identifies all changes in chromatin landscape. Thus, it is uniquely suited for unbiased analysis of changes during differentiation. This powerful approach has recently been used to characterize adipocyte and osteoclast differentiation [15], [18]. Here we have applied DHS-seq to interrogate genome-wide changes in the chromatin landscape during MSC differentiation into OB.

A human cell line derived from fetal bone, hFOB 1. 19, was immortalized by temperature-sensitive SV40 pUCSVtsA58 vector [19]. These cells were recently found to have broad MSC characteristics with the capacity of differentiation to OB, adipocyte and chondrocyte lineages, a process initiated by suppressing SV40 t-antigen expression at non-permissive temperature [20]. This “spontaneous” process is inefficient and takes several weeks (ibid). To obtain information on known and novel chromatin changes that lead to transcriptional regulation during OB differentiation, we compared control cells to cells undergoing spontaneous differentiation and with the effect of Osteogenic Induction Media (OIM). Multiple methodologies were employed, including global chromatin landscape profiling by DHS-seq and development of novel bioinformatic analysis to link these changes to mRNA expression. Our data is providing the first unbiased analysis of global chromatin remodeling and reveals novel potential mechanisms involved in early MSC commitment to OB differentiation.

## Materials and Methods

### Cells

Human fetal osteoblastic cell line, hFOB 1.19 (thereafter addressed as hFOB), was obtained from American type culture collection (ATCC, Manassas, VA, USA). This line was established from a fetal limb tissue and immortalized using SV40 pUCSVtsA58, a temperature sensitive expression vector with neomycin resistance gene, pSV2-neo [19]. Cells were maintained at 34°C in a 5% CO_2_ humidified incubator in Basal Media Eagle (Sigma, St. Louis, MO, USA, Cat# B1522) supplemented with 10% heat inactivated Fetal Bovine Serum (hiFBS), 0. 1mM non-essential amino acids, 1mM L-glutamine and 1mM pyruvate (all from Invitrogen, Carlsbad, CA, USA), thereafter addressed as BM. The media was also supplemented with 0. 3 μg/ml G418 every other passage. These cells divide every ~ 36hr with change of media every 3–4 days and passage every 8–10 days. Cells were trypsinized and plated for experiments when reached ~70–80% confluence.

### Differentiation Protocols

**Osteogenic induction:** was induced in cells at 80% confluence by shifting the plates to non-permissive temperature of 39°C for spontaneous differentiation in either BM or in media supplemented with Osteogenic Induction Medium (OIM, containing 100nM Dexamethasone, 100μM Ascorbic Acid, and 100μM β-GlyceroPhosphate, βGP). For assessing gene expression and bone matrix deposition, cells were plated in 60mm tissue culture plates at 1. 5x 10^6^cells/plate or in 24-well dishes at 0. 75x10^6^ cells/well for 24 hours and shifted to 39°C in 5% CO_2_ for indicated times. Media was changed every 3–4 days. Cells were harvested and compared to cells continuing to grow at 34°C in BM.

**Adipogenic induction:** was induced by shifting cells to 39°C in media supplemented with Adipogenic Induction Medium (AIM, containing 1μM Rosiglitazone, 0. 45μM IBMX, and 2. 5μM Insulin). Media was changed every 3–4 days. AIM was also supplemented with 100nM Dexamethasone for the first 3 days of induction. Cells were plated at the same density as above, harvested at indicated times and compared to cells growing at 34°C.

#### Alizarin Red S staining

Osteogenic differentiation was assessed by the appearance of extracellular matrix deposition using Alizarin Red S stain [8] (Sigma, St. Louis, MO, USA, Cat# A5533) according to the manufacturer’s instructions. Briefly, cells were fixed directly in media using 1:2 dilution of 8% (4% final) PFA in PBS for 15min at room temperature. Cells were washed twice with PBS and stained using 0. 1% Alizarin Red S stain in nuclease-free H_2_O at room temperature for 15min, followed by washing twice with H_2_O. Cell staining was observed and photographed using an inverted microscope at 10-20X magnification.

#### Oil Red O staining

Adipogenic differentiation was assessed by the presence of intracellular oil droplet formation using Oil Red O stain [8] (Sigma, St. Louis, MO, USA, Cat# O0625) according to the manufacturer’s instructions. Briefly, were fixed directly in media using 1:2 dilution of 8% (4% final) PFA in PBS for 1hr at 4°C and washed twice with PBS. Cells were then stained using Oil Red O Working solution (2.7mg/ml Oil Red O in 6:4 isopropanol: water solution) for 1-2hrs at 4°C, and washed twice with PBS. Oil droplet formation was visualized using an inverted microscope and photographed at 10–20 magnification.

### Cell cycle analysis

To examine cell cycle during differentiation, we used propidium iodide (PI) staining and FACS analysis. In brief, 1–2 x 10^6^ cells were trypsinized, washed with PBS, fixed in 1% PFA for 15 min at room temperature, and washed again with cold PBS. Cells were resuspended in 70% ice-cold ethanol and stored at -20°C until needed. After removing ethanol, cell pellets were resuspended in 50ul RNAse (100μg/ml) for 2hr at room temperature, washed 2X in PBS, and stained with PI (50μg/ml). Cells were filtered using 40uM filter and analyzed by FACS (FACS Calibur, Becton Dickinson).

### Gene expression analysis by qPCR

Total RNA was extracted from intact cells directly on the tissue culture plate using Trizol reagent with RNAeasy Mini Kit (Qiagen) at indicated times. RNA concentration and quality was measured using Nanodrop (ND 1000Spectrophotometer). cDNA was synthesized from 2ug of total RNA using iScript cDNA synthesis Kit (BioRad, Hercules, CA, Cat# 170–8891). Real-time PCR was performed in duplicate samples using iQ SYBR Green Super Mix (Biorad Hercules, CA, Cat#170-8882) for 45 cycles in the Light Cycler 2. 0 PCR system (Roche Diagnostics, Hercules, CA). mRNA levels were normalized to human βActin (S1 Table). Statistical data was compared using at least 3 independent biological replicates.

### Expression profiling

Total RNA (3ug of RNA per condition and time point; triplicate samples for each condition) was collected as above and processed and analyzed at NCI-Frederick core facility. Affymetrix microarray (Hu- U133 Plus 2. 0; Affymetrix, Santa Clara, CA) was used, providing comprehensive analysis of genome-wide expression on a single array. This microarray chip comprises more than 54,000 UniGene clusters derived from Build 95 of UniGene that analyze the expression level of more than 47,000 transcripts including 38,500 well-characterized human genes. Signals from each array were analyzed, normalized, and converted to a numerical output using Affymetrix GeneChip software. The average expression value for each gene across the arrays from triplicate samples was used to normalize the mRNA intensities. Relative ratios were obtained from the intensity value at each time point over the average intensity across the time course. The relative expression values were plotted as log2 of the ratio to facilitate visualization of quantitative changes between the time points. Heatmap using expression of top 135 genes with at least 2-fold change in expression taking into account at 3 independent experiments was plotted using heatmap. 2 in R program. Genes were annotated using Ingenuity Pathway software (http://www.ingenuity.com/) for cellular functions during differentiation and interactions and were mapped according to their instructions.

### Dnase I hypersensitivity analysis followed by deep sequencing (DHS-seq)

Cells were expanded in BM at 34°C to obtain ~80–100 x10^6^ cells/condition/each experiment and finally plated on 150mm dishes for 24–48 hours. Cells were continued to grow at 34°C or shifted to 39°C in BM or OIM for indicated times. DHS protocol was performed as previously described with minor modifications [21]. Briefly, nuclei were prepared in Buffer A (15mM Tris, 15mM NaCl, 60mM KCl, 1mM EDTA, 0. 5mM EGTA, 0. 5mM Spermidine) using 0. 02% NP40 followed by extensive washing in Buffer A and digestion at 37°C in a buffer containing 60mM CaCl_2_ and 750mM NaCl. Increasing concentrations of Dnase I from 0 to 100U/ml was added for 3min. The reaction was stopped with equal volume of Stop buffer containing 50mM Tris, pH 8. 0, 100mM NaCl, 0. 1% SDS, 100mM EDTA containing 50μg/ml Proteinase K (Invitrogen, CAT# AM2546). Samples were heated to 55°C for 3-4h and RNase I (25ug/ml, Roche, CAT#11119915001) was added for an additional hour. Dnase I digestion for each concentration of the enzyme was assessed on an aliquot of each sample by a Titration of Digestion (TOD) assay, a quality control protocol (thereafter addressed as TOD DHS-seq, see S1 Methods), and a single sample from each experiment with 70–80% digestion at common HS sites was selected for sequencing and generation of replica concordant (RC). DNA fragments were separated on a sucrose gradient (9% sucrose in 20 mM Tris HCl, 1 M NaCl, and 5 mM EDTA) using Beckman Ultra Centrifuge (Rotor SW41), and 50–400 bp fragments, identified on 2% agarose gel, were precipitated and sent for deep sequencing on Illumina GA2x sequencer at the Advanced Technology Center (ATC), National Cancer Institute (NCI-Frederick, MD, USA) using HiSeq2000 TruSeq V3 chemistry. All samples were good quality, with over 90% of the bases having Q30 or above, and yielded above 15 million reads. The library was measured by uniquely aligned reads using Picard’s mark duplicate utility.

### Deep sequence analysis by annotation to the human genome and assignment of hotspots

Biological replicas were selected by TOD DHS-seq method as described above, sequenced, and data were mapped to the reference genome hg19. Regions of local enrichment were identified using 36-mer short-read tags by DNase-seq by tools as previously described [22]. All samples had library coverage with 60–72% percent non-duplicated reads. Each tag was assigned to individual base pair to generate Maximal Density values (MaxD) value that represents the number of tags in each DHS. The tag density values were normalized to 10 million reads to adjust for the sequencing depth between replica samples. Replicate concordant (n = 2) of peaks in DHS [23] were identified among biological replicas as previously described [15] [22] with false discovery rate of 0%. Promoter regions were defined as sequences 2.5kb up and downstream from transcription start site (TSS). Regions of local tag enrichment were grouped as hypersensitive regions (HS). Exons and introns HS were assigned to the nearest TSS giving the priorities to the boundaries of promoters>exons>introns, with the remaining sequences assigned accordingly to distal up or downstream genomic regions. All sites modified were defined as a HS or those sites with significant differences (p<0.05) in MaxD. Sequencing data and accession link are summarized in S6 Table.

### De novo motif Analysis

To identify potential TF binding sites, we selected top 1,500 unique DHS sites (defined by changes in Max Density). We used MEME (-minw 6 -maxw 20) [24],and HOMER [25] (-size 200 -mask -len 6,8,10,12,14,1a6 -S 10 -bits) motif discovery software analysis (http://homer.salk.edu/homer/ngs/). As dictated by the maximal number of searches allowed for each analysis, we limited the search to 200 bp windows that spanned the hotspots within +/- 1KB of TSS. Matches were considered significant if the majority of sequence nucleotides were shared and P values were < 10^−4^.

### Computational analysis linking changes detected by DHS-seq to gene expression

To correlate changes in global chromatin landscape with gene expression during osteogenic differentiation, we adapted and modified bioinformatic analysis used by Ingenuity Pathway Analysis (IPA, http://www.ingenuity.com/) in two ways. First, we selected DHS sites +/- 2.5kb of TSS that were unique to each condition thus underwent significant change, appeared or disappeared at any time during differentiation. The second was to exclude the promoters, combining exons, introns and intergenic regions and these were designated as enhancers. These unique sites were paired with all genes that had TSS within 50kb to the border of DHS. To upload into IPA program, the differences in MaxD were converted into Intensity. Annotations of these gene lists by IPA generated plausible pathways affected by chromatin modifications that were unique to each condition. To correlate these pathways to gene expression, genes encompassed in these pathways were overlaid by gene expression obtained from microarray analysis.

## Results

### Differentiation of hFOB cells into OB and adipocyte lineages

**Fig 1:**
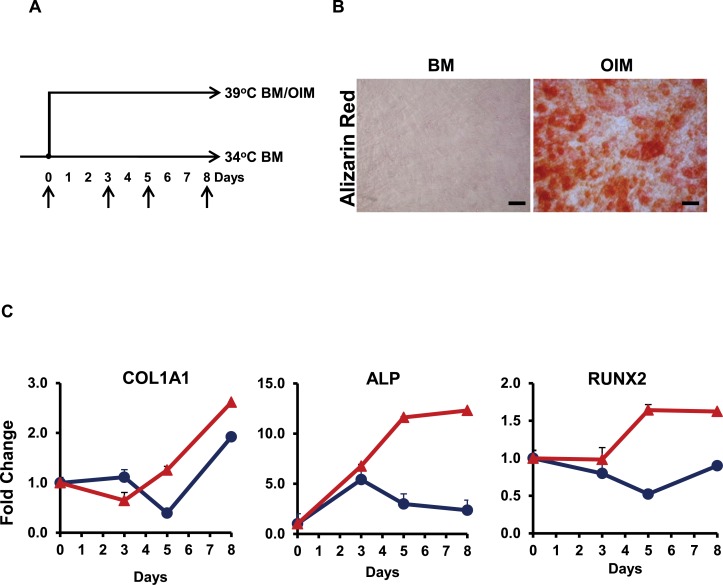
Time course of differentiation into osteoblastic lineage. (A) Schematic representation of the conditions. hFOB cells were expanded and maintained at 34°C in basal media (BM), thereafter designated as control cells. Cells were shifted to 39°C in BM or in Osteogenic Induction Media (OIM). Samples were collected at times indicated by the arrows. (B) Alizarin Red S staining of extracellular matrix deposition in control cells (left) and cells grown in OIM (right) for 8 days. Scale bars indicate 10 μm at 20X magnification. (C) Expression of OB related genes by qPCR in cells exposed to BM (blue) or OIM (red) at 39°C on days 0, 3, 5 and 8. The panels correspond to Collagen 1A1 (COL1A1), Alkaline Phosphatase Liver/Bone/Kidney (ALP), and Runt-related transcription factor 2 (RUNX2). All values were normalized to control cells and SD calculated based on triplicate samples. One of more than 3 independent experiments is shown here.

Proliferating hFOB cells at 34°C in BM were shifted to non-permissive temperature resulting in shut-down of t-antigen to induce spontaneous or OIM/AIM-induced differentiation for up to 8 days as indicated in Fig 1A. Cells exposed to OIM for 8 days accumulated significant bone mineralized matrix as detected by Alizarin Red S, whereas control cells growing at 34°C and cells at 39°C in BM had no evidence of matrix deposition at the same time (Fig 1B). We tested expression of multiple osteoblast-related genes: collagen type 1 alpha 1 (COL1A1), alkaline phosphatase (ALP), and runt-related protein 2 (RUNX2) as shown in Fig 1C. Cells exposed to OIM had a significant increase in expression of these genes which was evident by day 3 of induction. These data indicate that the exposure to OIM induces a rapid and more efficient osteogenic differentiation. To determine whether hFOB cells transiently become MSC-like when shifted to non-permissive temperature as previously reported [19], we also induced differentiation of these cells into adipocytes by Adipogenic Induction Media (AIM) for up to 8 days. Cells exposed to AIM had induction of fat-specific genes, such as PPARγ, and a robust adipogenic differentiation with accumulation of fat droplets detected by Oil Red O stain (S1 Fig).

Cells induced to differentiation at non-permissive temperature had an immediate decrease in proliferation, although there was no difference in cell number with or without OIM for 48 hours (data not shown). To test whether OIM media specifically contributes to changes in cell cycle, we examined the cell cycle in each experimental condition using Propidium Iodide (PI) staining. We found a brisk decrease in S-phase that was evident within 16 hours after shift to 39°C, with a corresponding increase in G2M and G0/G1 (S2 Fig). Notably, there were no significant differences between cells shifted to 39°C in BM or OIM. Thus, the cell cycle responds to the shift to 39°C, and reflects the lack of proliferative driving force provided by SV40. This was an important finding in assigning the role of OIM in specific pathways during osteogenic differentiation.

### Selection of biological replicas for sequencing based on Titration of Digestion (TOD-DHS)

To characterize changes in global chromatin landscape during spontaneous or OIM-induced OB differentiation, we employed DHS-seq analysis at early steps of commitment, at 24 and 48 hours (d1 and d2) after cells were shifted to non-permissive temperature with and without OIM. This was performed to normalize DHS-seq for samples collected at different days of differentiation and assure reproducibility of the results. To establish a method for selection of samples with comparable degrees of digestion, we harvested cells at different times and treated nuclei using a wide range of Dnase I. Based on these experiments, we developed a protocol which we named Titration of Digestion (TOD-DHS), a quality control step for selection of biological replicas for sequencing. We used a fraction of Dnase I digested DNA to perform qPCR using primers from common hypersensitive or resistant DHS sites derived from 48 mammalian cells in the large-scale epigenome mapping by the NIH Roadmap Epigenomics Project, the ENCODE Consortium (http://www.genome.gov/encode/) (S3 Fig and S2 Table). From these data and multiple additional experiments (S4 Fig and data not shown), we concluded that ~70–80% digestion at common hypersensitive sites represents an optimal sample that has the consistency for use as a biological replicas (S5 Fig). We used these parameters for selecting biological replicas for samples collected at different times of differentiation for all subsequent experiments. Bioinformatic tool for generation of Replica Concordant (RC) is described in Methods. Thus, all data presented is derived from RC on biological replica samples selected using TOD-DHS.

### Global Chromatin Landscape Transitions during OB Differentiation

**Fig 2:**
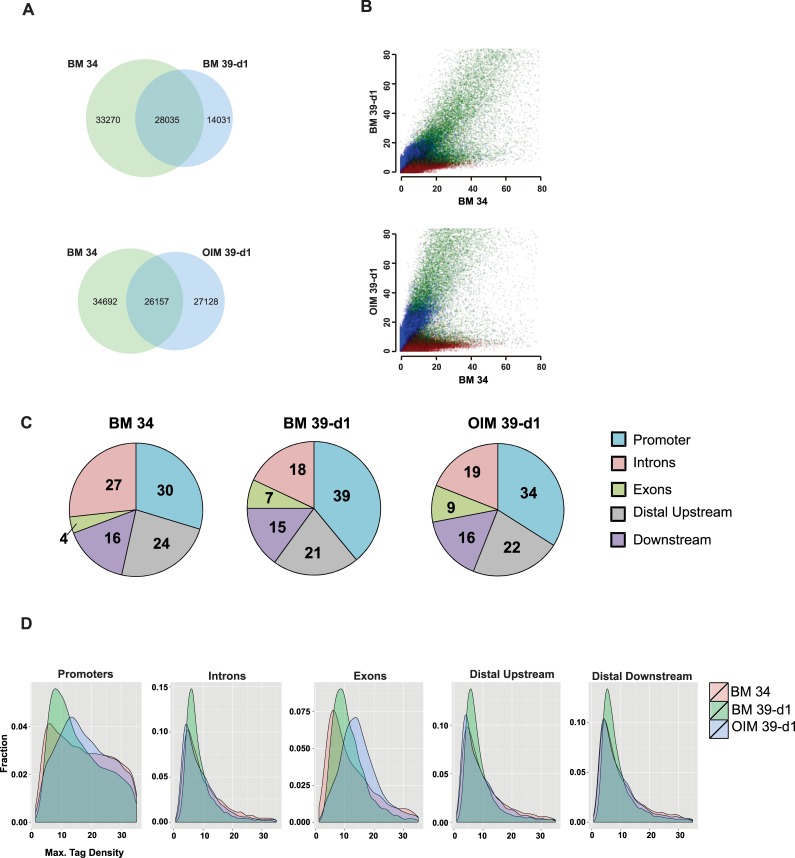
Comparison of Dnase I hypersensitive sites during differentiation. (A) Venn diagram representing pair-wise comparison of DHS among cells in BM39 d1 (top) and OIM d1 (bottom). The overlap represents commons sites identified by DHS, and the increase in unique sites in OIM is evident on the lower diagram. (B) Scatter plot representing the analysis shown in Venn diagrams. (C) Percentile of Tag densities in each condition representing global distribution of DHS in the promoter (blue), intron (pink), distal upstream (gray), downstream (violet) and exon (green) regions. (D) Aggregation plots from 3-way comparison of DHS tags separated by each region. The X-axis indicates MaxD, i. e. amplitude of DHS tags as described in Methods, and the Y-axis represents the fraction for each group of tags. Control cells (pink), cells in BM at 39°C (green) and in OIM (blue). All data shown are DHS sites from replica concordant using 2 independent biological replicas from 3–5 independent experiments.

To investigate changes in global chromatin landscape during OB differentiation, DHS-seq was performed in control cells growing at 34°C and in cells at non-permissive temperature (39°C) in BM or OIM for 1 and 2 days. The Venn diagram in Fig 2A represents that within 24 hours cells have a massive shift in DHS profile when allowed to undergo spontaneous differentiation in BM (14,031 unique sites, upper panel), and even greater when exposed to OIM (27,128 unique sites, lower panel). Scattered plots for each condition show increasing differences for each pair comparison (Fig 2B). The changes in DHS distribution indicate novel chromatin modifications that are transiently or permanently open or closed, and are likely to contain regulatory sites. We then examined genome-wide distribution of all DHS sites in each condition. Promoters constituted ~30% of total DHS and their fraction increased during differentiation (Fig 2C). The remaining sites were divided among downstream, distal upstream and intron regions, with exons representing the smallest fraction. However, the fraction corresponding to the exons had the largest change, from 4 in control cells to 9% in OIM. Further analysis of each fraction on d1 is visualized in aggregation plot (Fig 2D). There were substantial differences in the promoters, especially with an increase in the amplitude of MaxD (number of tags per site, X-axis) in OIM treated cells, although B39 cells had higher fraction of DHS with lower MaxD values (Y-axis). Higher average MaxD were also observed in exons of OIM treated cells. Note that the averaged tag densities were similar across DHS in distal upstream and downstream sites. Similar findings were evident for each time point at day 2, with further increase in MaxD in OM treated cells specifically in promoters and exons (data not shown).

**Fig 3:**
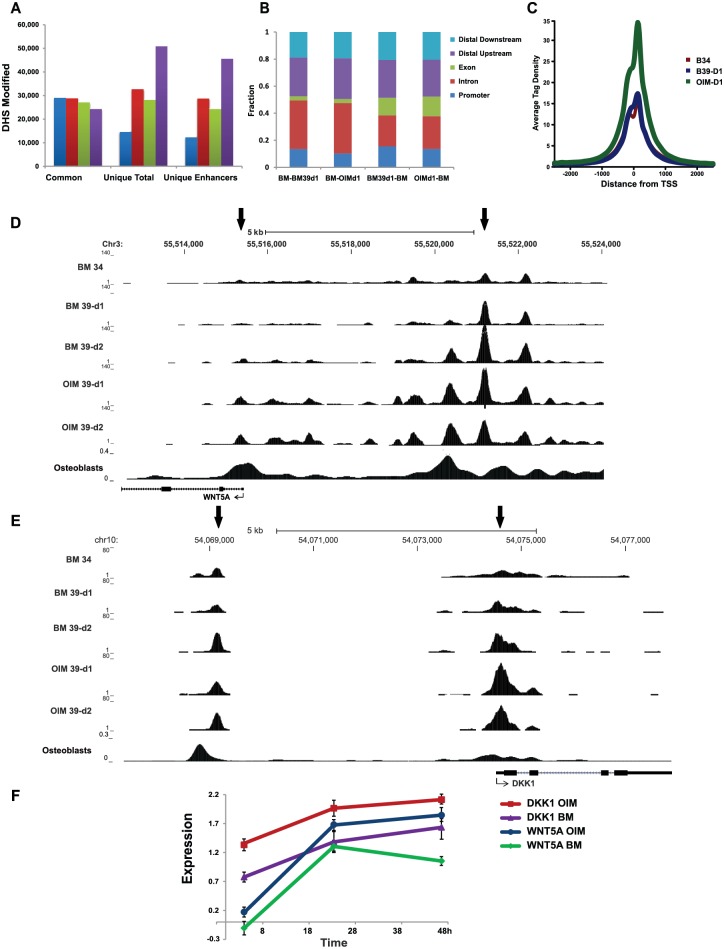
Analysis of Dnase I hypersensitive sites during OB differentiation. DHS sites in cells shifted to 39°C in BM or OIM for 24 and 48hr (d1 and d2) were compared to control cells growing in BM at 34°C (BM 34). (A) Total number of DHS sites from 2-way comparison as indicated by colors on the right of the bar graph (blue: B39d1-B34, red: B39d2-B34, green: OIMd1-B34, purple: OIMd2-B34) was separated into common, unique and unique enhancers, designated as sites after removing the promoters. (B) Ratio of unique DHS sites separated into promoters, exons, introns, and distal up and downstream sites. The first 2 bars represent BM control cells compared to differentiating cells with no significant shift of fractions. The last 2 bars show the reverse comparison, using DHS unique for BM39 and OIM. (C) Aggregation plots showing average tag density relative to TSS from 3-way comparison with significant increase in MaxD from OIM treated cells. (D) An example of DHS tracing for WNT5A locus (UCSC browser, [27]) show changes in the proximal enhancer located upstream within 5kb of WNT5A TSS. Chromosomal coordinates and scale are indicated on the top. Arrows indicate sites changed between control and treated cells. Each condition is annotated on the left of each tracing. DHS for mature bone osteoblasts is added for comparison (from ENCODE database, Short Read Archive: GSM816654). d. (E) DHS tracing for DKK1 locus shows changes in the promoter. Designations are as above. Each tracing represents replicate concordant from 2–4 independent experiments. DHS for mature bone osteoblasts is added for comparison (from ENCODE database, Short Read Archive: GSM816654). (F) Expression changes in WNT5A and DKK1 showing log2 values from 3 biological replicas. Color labels are indicated on the right.

We then examined the total number of sites that were common or unique using 3-way comparison between control and cells in BM or OIM. OIM-treated cells had 3–4-fold higher number of unique chromatin modifications at all time-points, as compared to cells in B39 (S6A and S6B Fig). In contrast to 2-way comparison between cells on d1 or d2, shown in Fig 2A and 2B, there were a larger number of common DHS sites among cells exposed to OIM for 1 day with cells in BM for 2 days, with fewer unique sites (S6C Fig). The total numbers of common and unique sites for each pair are presented as a Table in S6D Fig These data further support the observation that a) OIM-induced differentiation has unique features, and b) it is delayed in cells exposed to BM at 39°C. This trend was further evident when comparing common and unique sites (Fig 3A) with unique sites more than doubled by d2 in OIM treated cells. This trend was maintained when the unique sites modified in the promoters were removed, and the remaining sites were designated as enhancers. In addition, DHS in cells in OIM on d1 were more like B39 cells on d2, with a sharp increase in common sites (S6C Fig).

To explore the contribution by each component to changes unique for each condition, we analyzed their respective proportions on d1 using pair-wise comparison (Fig 3B). When comparing control to differentiating cells, no major changes were observed among each fraction (first 2 sets of bars). In contrast, there was an increase in unique DHS modified in the promoters of differentiating cells (last 2 bars). In addition, there was a substantial increase in unique DHS corresponding to exons, mostly at the expense of a decrease in introns. These sites could represent changes in transcription or alternative splicing. Thus, early steps of differentiation involve transcriptional regulation of genes with active regions in the promoters, but also a substantial fraction of change corresponds to the exons and introns. Previous studies using adipogenic differentiation of murine cells also showed an increase in promoter regions within 24 hours of differentiation [26], but that study did not provide detailed analysis of each component.

We then asked whether there were quantitative differences of uniquely modified sites in the promoters. A closer look at the promoters using aggregation plot, centered at TSS, showed a significant increase in average tag density specifically in OIM treated cells (Fig 3C). Examples of changes in DHS profile surrounding the promoters (UCSC browser shot [27]) are shown for CDH11 (osteoblast-specific cadherin) and a bone-related gene, COL1A1 (S7A and S7B Fig). Significant DHS changes in cells exposed to OIM appeared within one day of differentiation and correlated with changes in gene expression (S7C Fig). The DHS profile for mature bone osteoblasts from ENCODE database are added at the bottom of each tracing and showed that some hypersensitive sites that appeared during differentiation are also present in mature osteoblasts. Of note, not all changes persist in mature osteoblasts indicating a dynamic nature of chromatin landscape. Modifications in WNT5A promoter were accompanied by major changes in a potential WNT5A enhancer site located within 5kb upstream of TSS (Fig 3D). In addition, significant changes were identified by DHS in the promoter of a WNT inhibitor, DKK1, specifically in OIM treated cells (Fig 3E). Expression level of WNT5A in response to OIM increased on d1 but declined in by d2, which correlated with induction of expression of DKK1 (Fig 3F).

An example of DHS-uncovered chromatin modifications in a promoter and a distal exon is shown for GDF6 (S8 Fig). GDF6 is a member of bone morphogenetic protein and TGF-beta superfamily of secreted signaling molecules, which is required for normal formation of bones in the limbs, skull, and axial skeleton. Mutation of this gene is associated with skeletal malformations in Klippel-Feil syndrome [28].

Expression of Liver/Bone/Kidney Alkaline Phosphatase correlated with changes in DHS in the promoter region on chromosome 1 (Fig 1 and data not shown). In contrast, two other Alkaline Phosphatase isoforms, ALPP and ALPL2, located on chromosome 2, had no change in DHS in the promoters or expression. However, there was a large DHS ~5kb downstream of ALPP in all differentiating cells, in an area that did not overlap with any other gene, and thus could represent an enhancer for a distant target (S9A Fig). We examined expression of all genes within 100kb of this site. Only one gene, DIS3L2, located ~ 50kb upstream of ALPP had a sharp change in expression, with no significant changes in its promoter (S9B Fig). DIS3L2 is a mitotic checkpoint gene that regulates degradation RNA, is associated with body height, and plays an important role in differentiating tissues [29]. Although these findings need to be corroborated by experimental data, it is likely that the new DHS downstream of ALPP represents a novel enhancer for a distant target, such as DIS3L2.

### Global identification of regulatory elements during OB differentiation

To identify key transcription factors (TF) that participate in OB differentiation, we analyzed TF DNA binding motifs enriched within 1kb of TSS. Among top DNA binding motifs we confirmed selection of TF known to be involved in OB differentiation, such as AP1 [30,31] JUND, and RUNX [32], [33]. Additionally, we identified several novel TF binding motifs including TEAD1, ZBTB3, ZNF711, and ATF3. These TF have not previously been implicated in early osteogenesis. Of note, RUNX binding motifs had high scores in cells exposed to OIM within 24 hours, whereas no RUNX motifs with high scores were evident in cells exposed to BM for 2 days (S3A and S3B Table). In spite of dexamethasone, a component of OIM, we did not detect glucocorticoid response elements (GREs) within the top enriched motifs at d1 or d2. Because hFOB cells are programmed to spontaneously differentiate into osteogenic lineage, GREs may play a lesser role in this system. In addition, a previous study showed that GREs are enriched at an earlier time, within 4 hours but decline afterwards in L1 mouse cells treated with adipogenic differentiation media that also contains dexamethasone [15]. Early commitment steps at 4 hours were not included in our present study.

### Analysis of gene expression

**Fig 4:**
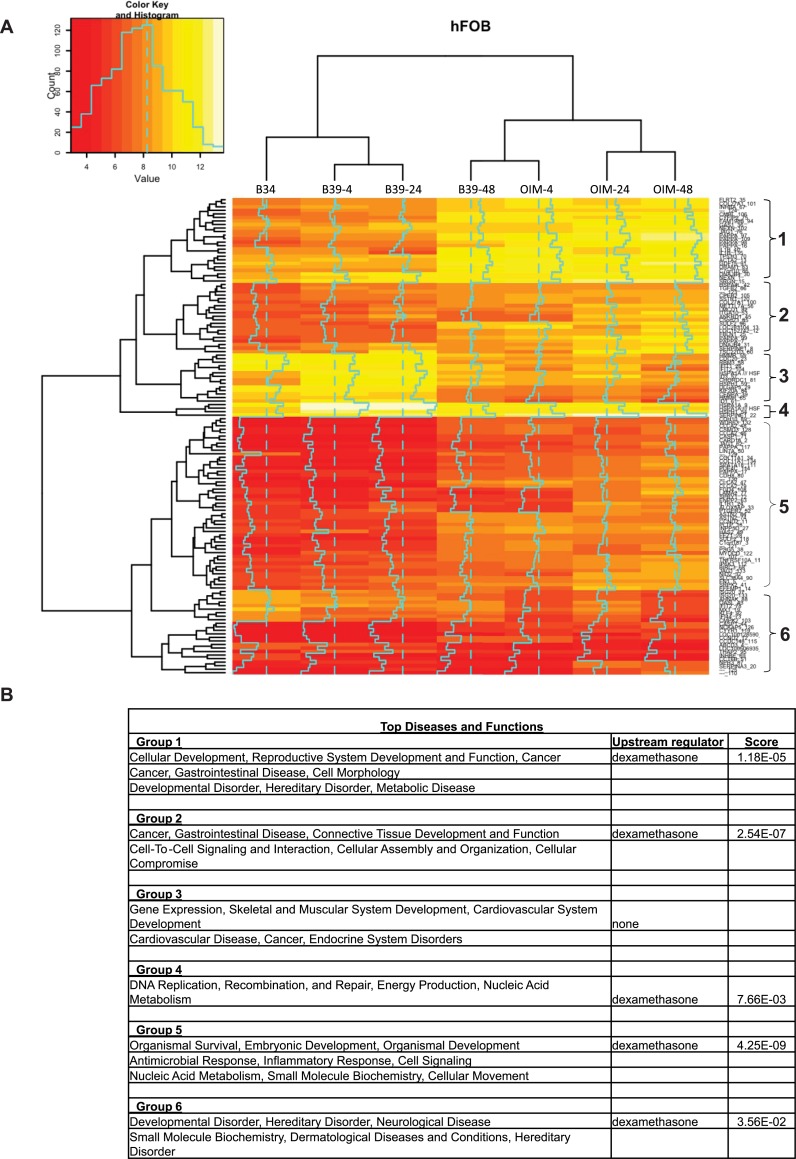
Heatmap of microarray analysis. (A) Heatmap of expression for top 135 genes with at least 5-fold change in expression was created using heatmap.2 in R program. These genes were separated into 6 major groups. Complete list of these genes with fold change can be found in S5 Table. Expression for each gene is calculated as difference from control values. All data represents means of at least three independent biological replicas. (B) Top networks identified by IPA represented by the 6 groups as shown in the heatmap. Dexamethasone was selected among several top upstream regulators indicating additional value of using this type of analysis. The scores for Dexamethasone were assigned by IPA program and is shown on the last column.

We examined gene expression by microarray analysis at 4, 24 and 48 hours in cells exposed to 39°C with and without OIM. When compared to control untreated cells, unsupervised analysis of top 135 genes whose expression changed more than 5 fold showed that samples segregated according to time and condition (Fig 4A and S4 Table). Hierarchical clustering showed 6 distinct groups of genes. Expression of genes in cells exposed to OIM for 4 hours was closer to those exposed to BM39°C for 2 days. These data correlated with the analysis of global changes in DHS and support the conclusion that OIM accelerated osteogenic program. Annotation of these 6 groups of genes by GO analysis showed that top functions were in developmental processes including skeletal, embryonic and organ development (Fig 4B). In addition, dexamethasone was assigned by IPA with relatively high scores as the major upstream regulator in 5 of the 6 groups.

### Correlation of DHS-seq with Gene Expression

**Fig 5:**
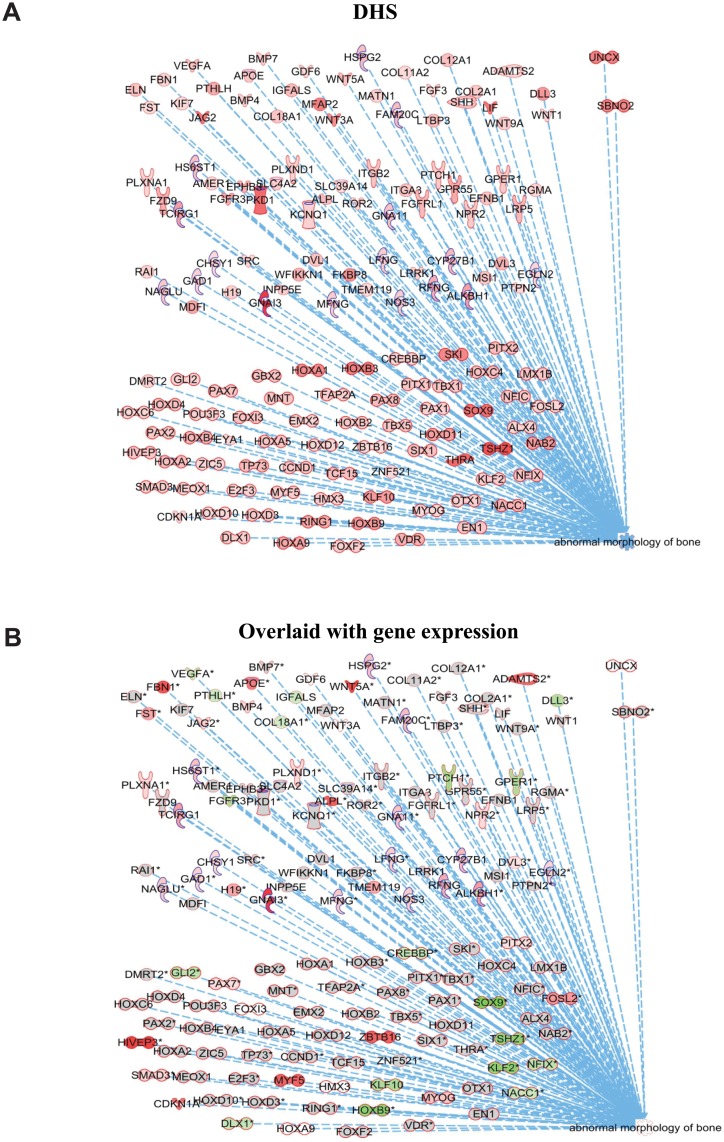
Linking DHS to gene expression. (A) Top pathway in OIM at d1 from unique DHS in enhancer regions (removing promoters) identified by IPA was Tissue development. Within that group a network of top genes was assigned to bone development, comprising 147 genes, with p <6.5x10^-19^. The graph shows subcellular localization of these genes with top section representing extracellular, followed by plasma membrane, cytosol and nuclear compartments. Colors represent the degree of DHS modification for each gene. (B) Same as above, with colors representing gene expression (overlaid by IPA from OIM compared to control cells from microarray analysis) at the same time point. The full gene list and changes in mRNA expression are shown in S5 Table.

It is desirable to link the information on changes in global chromatin landscape assessed by Dnase I hypersensitivity to alterations in gene expression and networks affected by these changes. There are no consistent programs to perform such comparison. To bridge the gap between these two sets of data, we modified the Ingenuity Pathways Analysis (IPA) program (described in Methods). All hot spots, unique to each condition, were paired to those genes whose TSS was close to the nearest DHS border. This gene list and genome-wide coordinates of DHS were annotated by the IPA knowledge base. Interestingly, analysis of enhancers unique to cells in OIMd1 as compared to B39d1, assigned the highest priority to “Tissue and organ development” with a subset of 147 genes within “abnormal bone development” (S5 Table) had the highest p value of 6.5x10^-19^. Network analysis of these genes (Fig 5A) shows the complex interaction of these gene products separated into “extracellular”, “plasma membrane”, “cytosol”, and “nuclear compartments”. The list of these genes, selected by IPA from our data set, was overlaid with mRNA expression from microarray at the same time points (Fig 5B). Most of the genes in this group also had significant changes in expression, as shown in S5 Table. Thus, it is possible to identify functional networks and specific genes from unbiased analysis of unique enhancers.

## Discussion

Our study is the first showing changes in global chromatin landscape during early steps of osteoblast differentiation, and providing a novel method for linking these changes to gene expression. We uncovered dramatic changes in global chromatin landscape and identified numerous novel regulatory elements involved in early steps of MSC commitment to OB lineage.

Cell culture systems are an important tool in understanding cellular and molecular mechanisms of differentiation. Although cultures of primary MSC may reveal a more accurate view of *in vivo* responses, their limited lifespan *ex vivo*, heterogeneity among donors and differentiation stages restricts their use for DHS analysis. The human fetal cell line hFOB is a suitable model because it allows separation between effects of OIM, which contains glucocorticoids and the non-treated, slow process of spontaneous differentiation. These cells, immortalized by ts-t-antigen, proliferate as fibroblasts at 34°C and acquire MSC characteristics upon shutting down t-antigen expression at a non-permissive temperature. Surprisingly, many laboratories report studies using these cells proliferating at 37°C, when t-antigen is only partially shut down, without a full effect on their MSC characteristics and subsequent differentiation [34], [35].

Our studies confirmed previous reports that these cells can differentiate at 39°C into OB and AD lineages in response to specific media components [20]. Thus, they transiently become MSC-like when shifted to non-permissive temperature. Analysis of chromatin modifications, as defined by DHS-seq data, showed that OIM accelerates the differentiation process. As expected, the shift to non-permissive temperature with or without OIM is accompanied by a decrease in proliferation. We established that there was no difference in proliferative rate and that there was a rapid arrest in cell cycle with depletion of S phase and a modest accumulation in late G2. This process is equally evident regardless of exposure to OIM, and thus is a result of the shut-down of t-antigen. In spite of similarities in the effect on cell cycle, there were significant differences in chromatin landscape modifications and gene expression in cells exposed to OIM as compared to cells in BM during 2 days of differentiation.

Gene expression analysis showed that under spontaneous differentiation at 39°C cells underwent a significant stress with alterations in cell cycle, DNA replication and repair. These genes continue to be dominant throughout 48 hours, with additional effects on genes involved in cell movement. In contrast, exposure to OIM led to alterations in embryonic and differentiation-associated genes (JAG1, SMAD3, TGFβ, and others), connective tissue and bone development (with up-regulation of RUNX2 and several collagen genes by d2) in addition to cell cycle genes. Hierarchical clustering using unsupervised analysis also separated cells exposed to BM and OIM, with cells in BM for 2 days positioned closer to cells in OIM for one day, confirming the accelerated response to OIM evident from the analysis of DHS. The larger group of genes (group 5) encompassed genes that regulate embryonic differentiation and organ formation. Interestingly, a smaller group of genes (group 4) encompassed DNA repair and recombination. Recently, TOPOI recruitment to DNA has been shown to be required for androgen-mediated activation of enhancers, and recruitment of other members of DNA repair machinery [36]. Thus, differentiation to OB lineage is also accompanied by increased expression of DNA repair genes. Furthermore, dexamethasone was identified as the top upstream regulator for group 5 with a score of 4x10^-9^, and had high scores in several other groups shown in Fig 4B, further indicating the value of the analysis performed by IPA.

Chromatin modifications assessed by DHS-seq showed massive and reproducible genome-wide modifications during differentiation, with significant differences between cells exposed to OIM and BM. The unique modifications uncovered by DHS for each condition represent areas that are remodeled (changes in tag number annotated as MaxD), open or closed during differentiation process. Not surprisingly, there were significant changes in promoter regions, which suggest that TF binding is highly engaged during this process. The search for specific TF motifs that were unique to each condition showed that the cells in BM have a preference for TFs which regulate cell cycle, whereas cells exposed to OIM as early as d1 preferentially induce modifications at sites that recognize RUNX and other OB related TFs, in addition to the above mentioned motifs.

An additional novel finding in our study is that >80% of unique chromatin modifications occur in genomic regions outside +/-2.5kb of TSS, encompassing exon, intron, intergenic and distant enhancer regions. Cells exposed to OIM for one day had over 16,000 sites (approximately 1/3d of all DHS) that were not overlapping with cells in BM, or with sites in cells growing at 34°C using a 3-way analysis, and thus were unique to OIM. A previous study from our laboratory reported that early stages of adipogenesis are associated with changes in the promoter and distal enhancer regions [15]. In contrast, we found that exons, representing the smallest fraction of total unique DHS, had the major changes, especially in OIM.

One of the difficulties in current understanding the functional outcomes of chromatin modifications is the lack of a method that can link all DHS sites to gene expression and networks they potentially modify. To bridge this gap, we assigned all nearest genes to unique DHS in enhancers (excluding the promoters) and used IPA analysis to identify specific pathways that involve these genes. This analysis allowed correlation of DHS changes to gene expression. Although the annotation of genes whose TSS is near any given DHS is somewhat artificial, the analysis using this approach identified tissue/organ formation and MSC differentiation as the top networks OIM differentiating cells. Furthermore, a subset of 147 genes that participate in bone formation, was identified with a p = 6. 5x10^-19^. The list of these genes included genes that directly or indirectly have been implicated genes in bone formation. Furthermore, when this network was overlaid with expression, most of these genes also showed a significant change in expression. This network also included modifications at NOTCH and WNT family members. Recent studies linked NOTCH and WNT, the two main signaling pathways driving oscillatory clock for mesodermal patterning, by WNT regulation of DLL in a mouse system [37]. In addition, WNT pathway is critical in OB differentiation and inhibition of WNT pathways is associated with significant bone abnormalities [38], [37]. However, changes in DHS surrounding the promoter and proximal enhancers for several members of WNT family, like WNT5A, were transient in OIM, and did not continue to increase at d2, a finding that correlated with gene expression. Furthermore, changes in DHS at WNT5A locus and gene expression were one of few examples where cells in BM for 2 days had higher values than did cells in OIM. These findings suggested that WNT induction and possibly expression were suppressed by prolonged exposure to OIM. Remarkably, we found that OIM induced a significant increase in DHS at the promoter of DKK1, a known competitive inhibitor of the WNT pathway whose expression is linked to osteopenia and bone metastasis [39], [40]. Furthermore, DKK1 promoter also contains glucocorticoid (GC) responsive elements [41] and its up-regulation is documented after prolonged exposure to GC [42]. We found that DKK1 has a 2-fold up-regulation in OIM treated cells. Thus, OB induction program may be blunted by exposure to GC, and development of an inhibitor for DKK1 may have potential therapeutic applications.

Although OIM also contains ascorbic acid, which has known mild antioxidant effects, and β-glycerophosphate, GCs are considered to be a major component for *in vitro* OB differentiation [30,31,33]. The molecular basis of GCs, transiently released during stress in response to bone fracture *in vivo*, have not been fully elucidated. Thus, using IPA program was instrumental in bridging global unbiased analysis of changes in chromatin landscape with specific information on gene expression. Previous reports indicated that a fraction of DHS modifications correlate with changes in nacent RNA [13], [15]. Here we provide a method by which chromatin modifications can identify specific networks, and correlate with changes in gene expression.

Previous reports indicate that the AP-1 family of TF, including FOS and JUN are involved in the early response to OB differentiation [31]. We found multiple DHS modifications at sites surrounding the promoter regions of these genes. In addition, substantial modifications occurred for JUN at 2 sites distant to the promoter, at ~20-100kb upstream of TSS (S10 Fig) with no other genes within vicinity of those sites. These genomic loci also have intense H3K27 acetylation and H3 mono-methylation, indicating active gene regulation in many cell types [43]. Thus, these sites may represent previously unrecognized enhancers for JUN. Whether these enhancers are operational in these or other cells, need to be validated experimentally.

In conclusion, we used DHS-seq to map open and closed chromatin sites on a genome-wide scale during early steps of OB differentiation and linked these changes to gene expression. Identification of these sites provided an unprecedented view of genome-wide transcriptional regulation during OB differentiation. Our analysis let to identification of functional networks of bone development using unbiased analysis of unique enhancers, and assigned dexamethasone as a major upstream regulator of changes in gene expression. Further studies will be necessary to verify experimentally which enhancers regulate specific pathways, and thus could be amenable for targeting to modulate bone formation and fracture healing.

## Supporting Information

S1 Fig hFOB differentiation into adipogenic lineage. To confirm that hFOB cells have MSC characteristics when shifted to non-permissive temperature, cells were expanded and maintained at 34°C in BM, then shifted to 39°C for differentiation in AIM for 8 days. Cells were fixed, stained with Oil Red O and oil droplets visualized on d8 using Nikon TS100 microscope. AIM contains dexamethasone 100uM during the first 2 days as described in Methods. Control cells (left) and in AIM (right) are shown. Scale bars indicate 10 μm at 20X magnification.
(EPS)

S2 Fig Changes in cell cycle during differentiation. Flow cytometry analysis of cell cycle during differentiation in cells stained with propidium iodide. Cells were shifted to 39°C and treated with OIM or BM39 for up to 72 hours. (A) Histograms showing the progression of the cell cycle after 48 hours of differentiation. Bars represent G_0_/G_1_(M2), S (M3), and G_2_/M (M4) phases in control cells (BM 34) and cells exposed at non-permissive temperature in BM or OIM. (B) Graphs representing the time course of changes in each cell cycle phase for each condition. Time 0 indicates cells growing at 34°C in BM. There was no statistical difference between cell cycle of cells in BM (blue) or OIM (red). There was a significant decrease in S-phase in both sets of differentiating cells within 16hrs with a corresponding ~7.5% increase in G_0_/G_1_ and ~4.5% increase in G_2_M. These changes are consistent with the effect of shutting down SV40 and are independent of OIM. Results of 3 independent experiments from a total of 5 are shown here, with means and SD indicated by bars.
(EPS)

S3 Fig Visualization of common Dnase I hypersensitive and resistant sites used for generation of the primers in 48 cell lines from ENCODE (http://genome.ucsc.edu/ENCODE/. Arrows indicate position of the primers for common positive and negative hypersensitive sites. Primers were selected from these sites (S2 Table).
(EPS)

S4 Fig Titration of Dnase I digestion (TOD-DHS) assay. TOD-DHS is an assay that evaluates the degree of digestion at common open and closed chromatin sites following Dnase I treatment. (A) Migration of nuclei treated with increasing concentrations of Dnase I (0-80U/10^6^ nuclei) on 1% agarose gel, showing a difficult task on deciding which concentration is best for sequencing. (B) Quantitation of the degree of digestion by qPCR using primers from common Dnase I hypersensitive sites within DCTN4 (dotted line) and EEF1A1 (solid line) genomic loci. The data is normalized to control (undigested) sample (Dnase I concentration = 0). The ordinate indicates fraction digested. Samples treated with 20, 40 and 80U of Dnase I were selected for deep sequencing for comparison (circled, X-axis). No digestion was seen using Neg1 or Neg2 primers (data not shown). (C) Venn diagram representing a pair-wise comparison of total sites shows a significant increase in DHS after 80 (blue) with an overlap with sites treated with 40 (pink) units of Dnase I, and few unique sites for 40U (orange). The total number of DHS increased almost 2-fold with increasing Dnase I concentration from 40 to 80U, with a small number (170 sites) being unique to 40U. (D) Table of three-way comparison of DHS sites in samples treated with 20, 40, and 80U of Dnase I. Common sites for each pair and individual unique sites are in the last column. (E) Changes in DHS tag tracing with increasing concentration of Dnase I viewed at *JUN* locus on Chromosome 1. The tracing shows significant increase in maximal density and appearance of new DHS at *JUN* promoter with increasing concentrations of the enzyme. Chromosomal coordinates and scale are indicated on the top. The arrow indicates TSS.
(EPS)

S5 Fig TOD-DHS analysis of Dnase I digestion. Cells were differentiated to osteoblastic lineage in BM or OIM at 39°C for 24 (d1) and 48 hours (d2). The upper panel of each section shows 1% agarose gel with migration of nuclei following Dnase I digestion (left) or sonicated nuclei (right) with variable fragmentation, but importantly no residual DNA band as on the left panel to allow extraction for qPCR. The graphs below the gels show fraction of DNA digested at one of the most sensitive sites for these cells within EE1A1 gene locus. SDs are for each point are calculated based on triplicate samples. Samples corresponding to 80% digestion (circled Dnase I concentration on the X-axis) were subjected to sequencing. One of 7 independent experiments is shown here.
(EPS)

S6 Fig Changes in Dnase I hypersensitive sites during differentiation. (A) Venn diagram from 3-way comparison of DHS mapped at d1 in cells shifted to non-permissive temperature (39°C) in BM or OIM, compared to control cells grown at 34°C. Differentiated cells showed a decrease in the number of common hypersensitive sites with corresponding increase in unique sites. (B) Venn diagram of all DHS on d2 as above. (C) Venn diagram in a 2-way comparison showing significant overlap of DHS sites between cells in BM 39 on d2 and OIM 39 on d1, larger than the common sites comparing cells on d1 and d2 shown in Fig 2A. (D) Table with the total number of common and unique DHS sites from 2-way comparison on d1 and d2 (left are unique sites for the 1^st^ and right for the 2^nd^ condition, respectively). Last lane highlighted by * is sown as Venn diagram in panel C.
(EPS)

S7 Fig Examples of changes in DHS profile at the promoters. (A) Modifications at the promoter of CHD11 locus. Top lane shows cells growing at 34°C in BM, followed by cells in BM at 39°C at d1 and d2, and cells in OIM at the same times. (B) Modifications at COL1A1 promoter, as above. Arrows indicate sites significantly changed between control and treated cells. Each tracing is the result of replica concordant obtained from 2 independent experiments. Chromosomal coordinates and scale are indicated on the top. DHS profile for mature osteoblasts from ENCODE Project Consortium, Sample Accession = GSM816654 is shown on the bottom of each panel. (C) Time-course of gene expression from microarray analysis (confirmed by qPCR, data not shown) as log2 changes. Colors and labels are indicated at the right.
(EPS)

S8 Fig Modifications in DHS sites at the promoter and an exon located >10kb from the promoter for SDF6 gene locus. Chromosomal coordinates and scale are indicated on the top. Arrows indicate sites changed significantly between control and treated cells in the promoter and first exon. DHS profile for mature osteoblasts from ENCODE database Sample Accession = GSM816654 is shown on the bottom. Each tracing is the result of replica concordant obtained from 2 independent experiments.
(EPS)

S9 Fig (A) An example of DHS downstream of ALPP gene locus representing a potential enhancer for a distant target. Chromosomal location and distance in kb are shown on the top. An arrow indicates the position of novel DHS sites ~5kb downstream from ALPP locus. (B) DHS profile at DIS3L2 promoter. An arrow indicates DHS site with variable tag density in differentiating cells that corresponds to a larger DHS in mature osteoblasts. DHS profile for mature osteoblasts from ENCODE database GEO Sample Accession = GSM816654 is shown on the bottom. Each tracing is the result of replica concordant obtained from 2 independent experiments. (D) Time course of ALPP and DIS3L2 expression during differentiation, showing that ALPP does not change and that expression of DIS3L2 is equally suppressed in BM39 and OIM.
(EPS)

S10 Fig Modifications in chromatin surrounding JUN gene locus. (A) DHS changes in the promoter of JUN with additional DHS sites detected >20kB upstream, representing a potential enhancer (B) Graphic representation of distances between JUN gene locus and 2 potential enhancers. (C) A closer look at the downstream proximal enhancer with DHS tracing amplified to 5kb. (D) A second potential enhancer detected ~100 kb in the same direction. No other known genes are found in the vicinity of these DHS sites. Below shown corresponding changes in H3K4Me1 and H3K27Ac from ENCODE database. DHS profile for mature osteoblasts from ENCODE database GEO Sample Accession = GSM816654 is shown on the bottom. Chromosomal location and distance in kb are shown on the top. Each tracing is the result of replica concordant obtained from 2 independent experiments.
(EPS)

S1 Methods (DOC)

S1 Table Primer sequences used for RT-qPCR assays for differentiation protocols. Abbreviations: ALPL—Alkaline phosphatase, liver/bone/kidney; ALPP—Alkaline phosphatase, placental; ALPPL2 —Alkaline phosphatase, placental like; COL1A1 —Collagen type 1 alpha 1; DLL1 —delta-like 1; DLL3 —delta-like 3; RUNX2 —Runt-related transcription factor 2; PPAR-γ —Peroxisome proliferator activated receptor gamma.
(PDF)

S2 Table Primer sequences used for TOD-DHS to standardize Dnase I treatment for selection of biological replicas. Primers were designed to overlap common hypersensitive or resistant sites to Dnase I selected from 48 human cell lines at ENCODE database (see S1 Fig). The column on the right indicates their genomic coordinates. ‘F’ denotes forward primer and ‘R’ reverse. Neg1 and Neg2 indicate sites resistant to Dnase I also shown on S1 Fig.
(PDF)

S3 Table A and B: Analysis of TF binding sites during differentiation. HOMER *de novo* motif discovery analysis of unique most frequently modified DHS within 1kb of TSS (http://homer.salk.edu/homer/ngs/) using 200 bp sequences spanning hotspots, revealed early RUNX involvement evident within 24 hours in OIM treated cells, while others, mostly cell cycle regulators, were present in BM cells at 39°C. No overlap indicates hotspots defining DHS sites that do not overlap with the comparable condition. (A) HOMER analysis of most frequent binding motifs using DHS sites unique for cells exposed to OIM as compared to BM39 for one day (upper panel, OIM_d1-B39_d1) or 2 days (lower panel, OIM_d2-B39_d2). (B) HOMER analysis of cells in basal media at 39°C compared to OIM on d1 (upper panel, B39_d1-OIM_d1) or on d2 (lower panel, B39_d2-OIM_d2). Note the presence of E2F and other motifs involved in cell cycle regulation in B39 for 24 and 48 hours, such as SPI-1 (PU. 1).
(PDF)

S4 Table List of top genes that changed expression during differentiation. Heatmap was constructed using expression of top 135 genes with >5- fold change in expression using heatmap.2 in R program. Expression for each gene is shown as difference from control values. All data represents means of triplicate biological replicas.
(PDF)

S5 Table Subset of 147 genes in “bone development” category identified by IPA from unique DHS enhancers on d1 OIM as compared to B39 as shown in Fig 5. This group of genes has a p value of 6. 5x10^-19^. Annotation for each gene was performed as described in Methods.
(PDF)

S6 Table List of all primary data deposited on Gene Expression Omnibus database, Short Read Archive: GSE75232. (PDF)
