# Supplementary material for: Genome-Wide Chromatin Landscape Transitions Identify Novel Pathways in Early Commitment to Osteoblast Differentiation
Source: PLoS One. 2016 Feb 18;11(2):e0148619. doi: 10.1371/journal.pone.0148619 (PMC4759368; doi:10.1371/journal.pone.0148619)
Supplement: S1 Methods — (DOC) [file pone.0148619.s011.doc]

S1 Methods

Development and Validation of Titration of Digestion for Dnase I Hypersensitivity (TOD-DHS). This test was established as a quality control for selection of specific sample from Dnase I treated nuclei preformed on different dates, in order to standardize the degree of digestion. We designed primers to known open or closed DNA sites that are common to human cells whose information is available from the ENCODE project (S3 Fig) ENCODE Project Consortium, <http://genome.ucsc.edu/ENCODE/>. The following procedure was used: a 100ul fraction of each sample digested with increasing concentrations of Dnase I was collected and sonicated to solubilize viscous DNA using Bioruptor300 (Diagenode, Denville, NJ, USA) and to allow isolation of total DNA. Sonication was performed for 6 cycles of 8-10 seconds each with 5-10 seconds rest in a water bath at room temperature on medium settings. DNA disruption was visualized on a 2% agarose gel, and if remaining undisrupted band was seen, additional 1-2 cycles of sonication were performed. DNA was isolated using QIAquick PCR Purification Kit (Qiagen, Valencia CA, USA, Cat#25106) and eluted in 50-100ul buffer as per manufacturer’s instructions. DNA concentration and quality was determined using Nanodrop1000^TM^. Ten ng of DNA from each sample was used for qPCR reaction using primers selected for common hypersensitive and resistant sites (described below, see S3 Fig and S2 Table). The data was normalized to control (untreated sample) for each primer pair.

Selection of primers for TOD-DHS. We obtained information on common Dnase I hypersensitive and resistant sites among 48 human cell lines from the large-scale epigenome mapping by the NIH Roadmap Epigenomics Project, the ENCODE Consortium (http://genome. ucsc.edu/ENCODE/). Three regions from common hypersensitive and 2 in resistant sites were randomly selected from the database (S3 Fig and S2 Table). Each primer pair was designed using Primer3Plus (<http://primer3plus.com/>) to obtain a product of 100–200bp (S2 Table). Multiple primers were subsequently validated in human cell lines for the best hypersensitive and resistant product obtained using increasing concentrations of Dnase I. To standardize the comparison between experiments, we selected samples with 70-80% of digestion by Dnase I at these sites for sequencing in all experiments described below (S4 and S5 Figs).
