# Supplementary material for: Genome-Wide Chromatin Landscape Transitions Identify Novel Pathways in Early Commitment to Osteoblast Differentiation
Source: PLoS One. 2016 Feb 18;11(2):e0148619. doi: 10.1371/journal.pone.0148619 (PMC4759368; doi:10.1371/journal.pone.0148619)
Supplement: S1 Table — Abbreviations: ALPL—Alkaline phosphatase, liver/bone/kidney; ALPP—Alkaline phosphatase, placental; ALPPL2 —Alkaline phosphatase, placental like; COL1A1 —Collagen type 1 alpha 1; DLL1 —delta-like 1; DLL3 —delta-like 3; RUNX2 —Runt-related transcription factor 2; PPAR-γ —Peroxisome proliferator activated receptor gamma. (PDF) [file pone.0148619.s012.pdf]

Supplemental Table 1

Gene transcript	Forward 5’→3’	Reverse 5’→3’
Reference Gene		
ß Actin	ACAGAGCCTCGCCTTTGCCG	TGGGCCTCGTCGCCCACATA
Differentiation genes		
ALPL (ALP)	GAACCCCAAAGGCTTCTTCT	GGGGTGTATCCACCAAATGT
ALPP	GGTGAACCGCAACTGGTACT	CCCACCTTGGCTGTAGTCAT
ALPPL2	ACAGCTGCCAGGATCCTAAA	CAAGCCAATGGTCTGGAAGT
COL 1A1	GAAAATGGAGCTCCTGGTCA	TAGCACCATCATTTCCACGA
DLL1	TGTGCCTCAAGCACTACCAG	GCGAGGTCATCAGGAGAATC
DLL3	TGAGCATGGCTTCTGTGAAC	CATTCAAAGGACCTGGGTGT
NOTCH1	ACTGTGGAGGACCTGGTGGAC	TTGTAGGTGTTGGGGAGGTC
RUNX2	CGGAATGCCTCTGCTGTTAT	TGGGGAGGATTTGTGAAGAC
PPARγ	TTCAGAAATGCCTTGCAGTG	CCAACAGCTTCTCCTTCTCG
