# Supplementary material for: Genome-Wide Chromatin Landscape Transitions Identify Novel Pathways in Early Commitment to Osteoblast Differentiation
Source: PLoS One. 2016 Feb 18;11(2):e0148619. doi: 10.1371/journal.pone.0148619 (PMC4759368; doi:10.1371/journal.pone.0148619)
Supplement: S2 Table — Primers were designed to overlap common hypersensitive or resistant sites to Dnase I selected from 48 human cell lines at ENCODE database (see S1 Fig). The column on the right indicates their genomic coordinates. ‘F’ denotes forward primer and ‘R’ reverse. Neg1 and Neg2 indicate sites resistant to Dnase I also shown on S1 Fig. (PDF) [file pone.0148619.s013.pdf]

Supplemental Table 2

Primer Name	Primer Sequence	Genomic Coordinates
EEF1A1_F	5’- TCTCTAGGCACCGGTTCAAT -3’	chr6:74287402-74287887
EEF1A1_R	5’- TCGTCATCACTGAGGTGGAG -3’	
DCTN4_F	5’- ATAGAGAACCCGGTCCGACT- 3’	chr5:150118621-150119016
DCTN4_R	5’- GGTGGGAAACCTAACGTCAA -3’	
USP5_F	5’- CGTTGCCGTTCTAAGCTAGG -3’	chr12:6831774-6832079
USP5_R	5’- CAGGTTTTCTCAAGGGTCCA -3’	
Neg1_F	5’- AGCCCTACTTTGGAAGCTGA -3’	chr12:36174942 -36174961
Neg1_R	5’- GGCATGAGGATTTCACTGGT -3’	
Neg2_F	5’- CAACAGTCCCCTGAGTGTGA -3’	chr2:35052543-35052793
Neg2_R	5’- AAATGTTGGGGATTTCTTGG -3’	
MYO1B_F	5’- GCCAGTTTCAGCTCACTTCC -3’	chr22:24468283-24468566
MYO1B_R	5’- CATGGAGGAGGAGCATTTGT -3’	
