# Supplementary material for: Genome-Wide Chromatin Landscape Transitions Identify Novel Pathways in Early Commitment to Osteoblast Differentiation
Source: PLoS One. 2016 Feb 18;11(2):e0148619. doi: 10.1371/journal.pone.0148619 (PMC4759368; doi:10.1371/journal.pone.0148619)
Supplement: S3 Table — HOMER de novo motif discovery analysis of unique most frequently modified DHS within 1kb of TSS (http://homer.salk.edu/homer/ngs/) using 200 bp sequences spanning hotspots, revealed early RUNX involvement evident within 24 hours in OIM treated cells, while others, mostly cell cycle regulators, were present in BM cells at 39°C. No overlap indicates hotspots defining DHS sites that do not overlap with the comparable condition. (A) HOMER analysis of most frequent binding motifs using DHS sites unique for cells exposed to OIM as compared to BM39 for one day (upper panel, OIM_d1-B39_d1) or 2 days (lower panel, OIM_d2-B39_d2). (B) HOMER analysis of cells in basal media at 39°C compared to OIM on d1 (upper panel, B39_d1-OIM_d1) or on d2 (lower panel, B39_d2-OIM_d2). Note the presence of E2F and other motifs involved in cell cycle regulation in B39 for 24 and 48 hours, such as SPI-1 (PU. 1). (PDF) [file pone.0148619.s014.pdf]

Supplemental Table 3A

OIM_d1 No Overlap B39_d1

Motifs	P-value	Best Match	Overlaps	
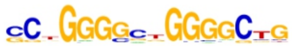	1.00E-14	**SP1**	**KLF4**	
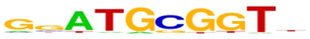	1.00E-14	**RUNX**	**RUNX1**	**RUNX2**
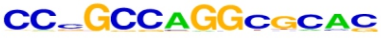	1.00E-11	**SMAD3**	**E2F1**	**E2A**
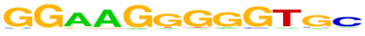	1.00E-11	**MAZ**	**SP1**	**SP4**
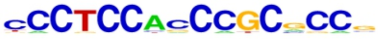	1.00E-10	**EGR**	**SP1**	**BCL6**
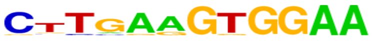	1.00E-10	**NKX2**	**STAT3**	
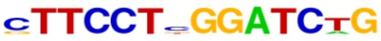	1.00E-07	**STAT3**	**SPI1**	**STAT6**
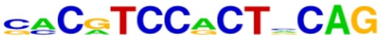	1.00E-06	**NKX2**	**SP4**	**ETS**
**OIM_d2 No Overlap B39_d2**				
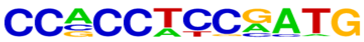	1.00E-12	**MAZ**	**RUNX**	**EGR**
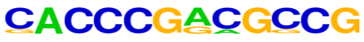	1.00E-12	**NR2F2**	**E2F**	**RARA**
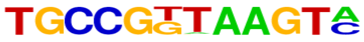	1.00E-12	**NKX3**	**STAT3**	
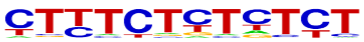	1.00E-11	**PRDM1**	**SMAD2**	**SMAD3**
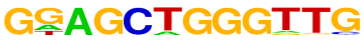	1.00E-10	**NHLH1**	**SP1**	**E2A**
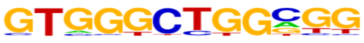	1.00E-08	**EGR2**	**EGR1**	**SMAD3**
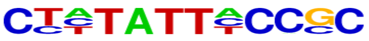	1.00E-08	**E2F1**	**E2F4**	**E2F6**
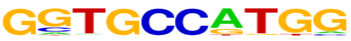	1.00E-08	**RFX1**	**E2F**	**YY1**
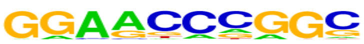	1.00E-08	**SPI1**	**RFX4**	**ETS1**
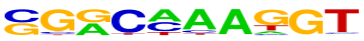	1.00E-08	**CEBPb**	**CRX**	**CEBPa**
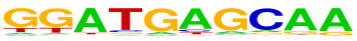	1.00E-07	**RXR**	**PPARg**	**TR4**
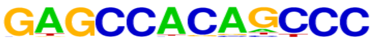	1.00E-07	**KLF4**	**RUNX1**	**RUNX2**

Supplemental Table 3B

B39_d1 No Overlap OIM_d1

Motif	P-value	Best Match	Overlaps	
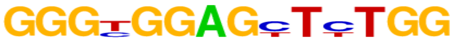	1.00E-14	GC	KLF4	BCL6
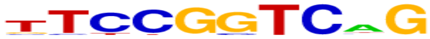	1.00E-11	ELK4	ETS	
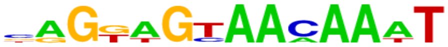	1.00E-10	FOXA2	SOX1	SOX4,5,6
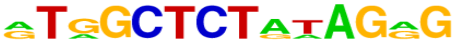	1.00E-08	TBP	DCE	E2F2
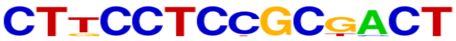	1.00E-08	SPI1	ETS	
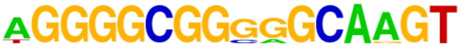	1.00E-06	E2F2	E2F3	
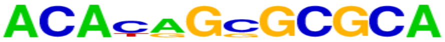	1.00E-06	KLF7	BCL6	SP1

B39_d2 No Overlap OIM_d2

Motif	P-value	Best Match	Overlaps	
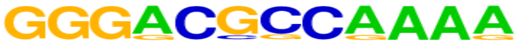	1.00E-14	E2F	E2F2	E2F3
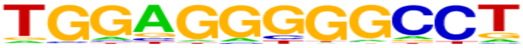	1.00E-12	MAZ	SP4	EGR
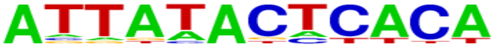	1.00E-12	SOX30	SRY	NKX3
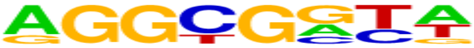	1.00E-11	ZEB1	E2F	EGR
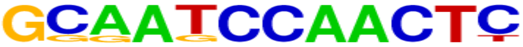	1.00E-11	EGR1	BRCA1	NFIC
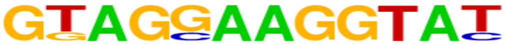	1.00E-11	SPI1	ETC	
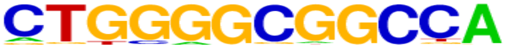	1.00E-11	BCL6	EGR	SP4
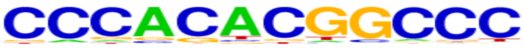	1.00E-10	PLAG1	SP1	EGR
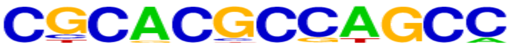	1.00E-09	ARNT	SMAD3	EGR
