# Supplementary material for: Genome-Wide Chromatin Landscape Transitions Identify Novel Pathways in Early Commitment to Osteoblast Differentiation
Source: PLoS One. 2016 Feb 18;11(2):e0148619. doi: 10.1371/journal.pone.0148619 (PMC4759368; doi:10.1371/journal.pone.0148619)
Supplement: S4 Table — Heatmap was constructed using expression of top 135 genes with >5- fold change in expression using heatmap.2 in R program. Expression for each gene is shown as difference from control values. All data represents means of triplicate biological replicas. (PDF) [file pone.0148619.s015.pdf]

	Gene.Symb	Gene.Title	B34	B39.4hr	B39.24hr	B39.48hr	OIM.4hr	OIM.24hr	OIM.48hr	diff_B39_4hr	diff_B39_24hr	diff_B39.48hr	diff_OIM.4hr	diff_OIM.24hr	diff_OIM.48hr	Visual Group
48	NEXN	nexilin (F a	8.9490066	9.8479000	10.254199	10.175766	10.454566	11.348700	11.419199	0.898893333	1.305193333	1.22676	1.50556	2.399693333	2.470193333	1
10423	ACTA2	actin, alpha	8.9347700	9.3420833	11.558666	11.216133	9.3354400	11.196833	11.385566	0.407313333	2.623896667	2.281363333	0.40067	2.262063333	2.450796667	1
11307	SRGN	serglycin	9.8528033	10.638779	11.642433	11.279700	10.580900	12.292000	12.566966	0.785976667	1.78963	1.426896667	0.728096667	2.439196667	2.714163333	1
11430	PAPPA	pregnancy-	6.4888200	7.3126266	10.071980	9.2642766	7.2979766	10.905600	11.432066	0.823806667	3.58316	2.775456667	0.809156667	4.41678	4.943246667	1
13258	DNAJB4	DnaJ (Hsp4	9.1359266	10.296333	10.316533	10.233566	11.055633	11.625366	11.803266	1.160406667	1.180606667	1.09764	1.919706667	2.48944	2.66734	1
13807	FLRT2	fibronectin	7.6769133	8.3947833	10.155799	9.7984633	8.5795066	9.7018800	10.374700	0.71787	2.478886667	2.12155	0.902593333	2.024966667	2.697786667	1
13905	GAS1	growth arr	8.7288433	8.0866833	9.4544333	9.7119466	9.3865199	11.183500	11.221200	-0.64216	0.72559	0.983103333	0.657676667	2.454656667	2.492356667	1
14515	IL1B	interleukin	8.5659066	8.2573400	11.853933	11.094099	6.9039366	9.4902700	11.092733	-0.308566667	3.288026667	2.528193333	-1.66197	0.924363333	2.526826667	1
19902	INHBA	inhibin, be	7.9560600	9.0458733	10.409666	9.8034433	8.5248733	10.102523	10.708166	1.089813333	2.453606667	1.847383333	0.568813333	2.146463333	2.752106667	1
19995	TP53I3	tumor prot	9.1752400	9.0554233	11.111199	11.284733	9.3784766	11.770466	11.783266	-0.119816667	1.93596	2.109493333	0.203236667	2.595226667	2.608026667	1
25079	CYFIP2	cytoplasmi	7.7090500	7.5517133	10.272633	10.125133	7.7283700	9.8507333	10.210099	-0.157336667	2.563583333	2.416083333	0.01932	2.141683333	2.50105	1
25561	JAG1	jagged 1	7.8661000	8.9409166	9.6818600	9.5237366	8.8661166	10.308233	11.649033	1.074816667	1.81576	1.657636667	1.000016667	2.442133333	3.782933333	1
27913	DRAM1	DNA-dama	8.9161000	9.0939500	11.629466	10.737566	9.0344300	10.448200	11.402933	0.17785	2.713366667	1.821466667	0.11833	1.5321	2.486833333	1
28940	C7orf10	chromosor	8.3743800	8.1355300	11.755766	10.988366	8.4690933	10.604733	11.663833	-0.23885	3.381386667	2.613986667	0.094713333	2.230353333	3.289453333	1
30860	GDF15	growth difl	9.4523333	9.2504733	12.060000	11.694133	8.9755333	11.419599	12.301499	-0.20186	2.607666667	2.2418	-0.4768	1.967266667	2.849166667	1
32483	FAM198B	family with	8.6080533	7.8929566	10.553966	10.362333	8.6024000	11.015533	11.219433	-0.715096667	1.945913333	1.75428	-0.005653333	2.40748	2.61138	1
34199	PAPPA	pregnancy-	7.2465566	8.3529933	11.067000	10.215366	8.4549933	11.829733	12.384966	1.106436667	3.820443333	2.96881	1.208436667	4.583176667	5.13841	1
34200	PAPPA	pregnancy-	6.4623633	7.7829666	10.400666	9.5678533	7.7768800	11.247500	11.753266	1.320603333	3.938303333	3.10549	1.314516667	4.785136667	5.290903333	1
34551	COL27A1	collagen, ty	7.6600300	8.4150133	10.094066	9.4764766	8.3131800	9.5674699	10.282133	0.754983333	2.434036667	1.816446667	0.65315	1.90744	2.622103333	1
35360	NEXN	nexilin (F a	8.2165066	9.2362433	9.4966066	9.3986866	9.6756333	10.573333	10.761933	1.019736667	1.2801	1.18218	1.459126667	2.356826667	2.545426667	1
36777	CMBL	carboxyme	7.0037300	7.5167666	10.492266	10.339066	7.9197433	10.667000	11.059166	0.513036667	3.488536667	3.335336667	0.916013333	3.66327	4.055436667	1
37383	PAPPA	pregnancy-	7.1897200	8.2886733	11.092733	10.203003	8.3417099	11.892200	12.429300	1.098953333	3.903013333	3.013283333	1.15199	4.70248	5.23958	1
47983	---	---	7.0864200	8.0335333	9.4839900	9.7264633	8.5077000	9.8330766	9.7763266	0.947113333	2.39757	2.640043333	1.42128	2.746656667	2.689906667	1
54330	IL1B	interleukin	8.4684800	8.0827133	11.564533	10.877000	6.2131533	9.3635533	10.843100	-0.385766667	3.096053333	2.40852	-2.255326667	0.895073333	2.37462	1
4764	PAPPA	pregnancy-	5.5438133	6.5487600	8.8156500	8.2575633	6.5892666	9.9077466	10.222930	1.004946667	3.271836667	2.71375	1.045453333	4.363933333	4.679116667	2
8844	SERPINE1	serpin pep	7.0033566	8.1979533	8.7974966	8.4274600	9.2143533	9.8375233	10.153396	1.194596667	1.79414	1.424103333	2.210996667	2.834166667	3.15004	2
12445	FBLN1	fibulin 1	6.6883066	6.7152700	9.2231000	8.1078466	6.9092433	8.8822600	9.7725366	0.026963333	2.534793333	1.41954	0.220936667	2.193953333	3.08423	2
13259	DNAJB4	DnaJ (Hsp4	7.2081300	8.6210000	8.3411700	8.4871900	9.4236066	9.9778133	9.8792533	1.41287	1.13304	1.27906	2.215476667	2.769683333	2.671123333	2
14991	HSPA4L	heat shock	6.2425700	7.8854466	8.6053900	8.5214633	7.8610633	8.5663033	8.3199033	1.642876667	2.36282	2.278893333	1.618493333	2.323733333	2.077333333	2
15477	ANKRD1	ankyrin rep	6.2262233	7.3300933	6.8613233	7.1273966	8.7273166	9.4078133	9.4832299	1.10387	0.6351	0.901173333	2.501093333	3.18159	3.257006667	2
16213	ITGA10	integrin, al	6.4505133	6.4544233	7.6942233	6.7571699	7.3015500	8.9690833	10.240516	0.00391	1.24371	0.306656667	0.851036667	2.51857	3.790003333	2
17203	METTL7A	methyltran	6.8768433	6.1027700	7.8238466	8.2591666	7.7526633	9.7666133	9.6288833	-0.774073333	0.947003333	1.382323333	0.87582	2.88977	2.75204	2
18179	TSC22D3	TSC22 dom	8.1412999	7.8045499	7.7725600	8.1922166	10.192000	11.149366	10.997633	-0.33675	-0.36874	0.050916667	2.0507	3.008066667	2.856333333	2
19316	TGFB2	transformi	6.1707433	8.0543000	8.4319533	8.3179233	7.6869300	8.5535700	8.5989933	1.883556667	2.26121	2.14718	1.516186667	2.382826667	2.42825	2
27860	LMCD1	LIM and cy	6.6162033	6.7129400	8.1372566	7.7351566	7.9866700	8.9401433	9.2893433	0.096736667	1.521053333	1.118953333	1.370466667	2.32394	2.67314	2
28339	C5orf23	chromosor	6.5978266	7.1088033	6.8379933	6.9846666	7.4427333	9.1581333	8.9639600	0.510976667	0.240166667	0.38684	0.844906667	2.560306667	2.366133333	2
33984	SULF2	sulfatase 2	6.7205499	6.1455033	9.7078566	9.4687399	6.6935000	9.8636133	10.079833	-0.575046667	2.987306667	2.74819	-0.02705	3.143063333	3.359283333	2
34201	PAPPA	pregnancy-	5.7917400	6.8845400	9.5786233	8.9076733	6.7912333	10.460400	10.937066	1.0928	3.786883333	3.115933333	0.999493333	4.66866	5.145326667	2
34546	COL27A1	collagen, ty	6.1889900	7.1130433	8.8562600	8.1150000	6.8911100	8.2208899	9.0016633	0.924053333	2.66727	1.92601	0.70212	2.0319	2.812673333	2
36195	CPEB2	cytoplasmi	6.6316233	7.8635966	8.6281733	8.4746266	7.8744333	9.1306933	9.2719733	1.231973333	1.99655	1.843003333	1.24281	2.49907	2.64035	2
44841	SSTR1	somatostat	6.5144333	7.5343533	9.0498900	8.6156799	7.2272733	8.2344733	8.9207666	1.01992	2.535456667	2.101246667	0.71284	1.72004	2.406333333	2
46685	---	---	5.8764900	7.7442300	8.4498900	8.4895766	8.3985766	8.8113900	9.0294833	1.86774	2.5734	2.613086667	2.522086667	2.9349	3.152993333	2
49417	LOC15274	hypothetic	6.6678933	6.7129233	9.1528500	8.5461533	6.9378966	9.2975599	10.390933	0.04503	2.484956667	1.87826	0.270003333	2.629666667	3.72304	2
52036	LOC28310	hypothetic	7.0623533	6.5281566	10.156303	9.8392800	6.4610066	8.4349799	9.4710833	-0.534196667	3.09395	2.776926667	-0.601346667	1.372626667	2.40873	2
14410	CENPA	centromer	9.7835033	10.066096	7.3313866	7.7904633	10.022813	8.1170366	7.2933233	0.282593333	-2.452116667	-1.99304	0.23931	-1.666466667	-2.49018	3
16612	HMMR	hyaluronar	11.328066	11.422633	8.7158566	9.3650166	11.215166	9.4636433	8.3949599	0.094566667	-2.61221	-1.96305	-0.1129	-1.864423333	-2.933106667	3
17266	ID3	inhibitor of	9.0490566	11.425966	8.7188433	9.6664666	11.658933	10.308133	8.4275766	2.37691	-0.330213333	0.61741	2.609876667	1.259076667	-0.62148	3
17744	RBM3	RNA bindir	11.748033	11.422499	8.6392466	8.5934633	11.359466	9.0135133	9.1707466	-0.325533333	-3.108786667	-3.15457	-0.388566667	-2.73452	-2.577286667	3
18160	HSPH1	heat shock	8.5480433	11.233466	8.9453300	9.3680233	10.962533	9.2377699	8.3776633	2.685423333	0.397286667	0.81998	2.41449	0.689726667	-0.17038	3
18353	ID1	inhibitor of	8.8217866	11.299433	6.2673966	7.8004133	11.563666	7.9568900	5.5903233	2.477646667	-2.55439	-1.021373333	2.74188	-0.864896667	-3.231463333	3
19118	HMMR	hyaluronar	9.6464599	9.8055799	6.8199200	7.5690633	9.5445100	7.6645233	6.5196400	0.15912	-2.82654	-2.077396667	-0.10195	-1.981936667	-3.12682	3
27852	CHORDC1	cysteine ar	8.4907166	11.057066	8.7718966	9.2546066	10.933233	9.3321666	8.8514000	2.56635	0.28118	0.76389	2.442516667	0.84145	0.360683333	3
28040	KIF20A	kinesin fan	10.307466	10.510533	7.5247366	7.9004833	10.367666	8.1128600	7.1760066	0.203066667	-2.78273	-2.406983333	0.0602	-2.194606667	-3.13146	3

36013	IFIT2	interferon-	10.783266	10.602133	7.8440266	10.006146	10.383466	9.7442300	6.9632133	-0.181133333	-2.93924	-0.77712	-0.3998	-1.039036667	-3.820053333	3
12030	HSPA1A //,	heat shock	9.2846333	12.096500	9.3060466	9.2676900	12.063300	9.3117933	8.7550533	2.811866667	0.021413333	-0.016943333	2.778666667	0.02716	-0.52958	3
12319	CDC20	cell divisior	11.068533	11.113733	8.2292000	8.9327200	11.134500	8.8754466	8.0084366	0.0452	-2.839333333	-2.135813333	0.065966667	-2.193086667	-3.060096667	3
12602	IFIT1	interferon-	10.801033	10.683500	8.1088033	9.9243366	10.324666	9.8996066	7.6222633	-0.117533333	-2.69223	-0.876696667	-0.476366667	-0.901426667	-3.17877	3
13212	DLGAP5	discs, large	10.454566	10.416466	7.6487966	8.2306833	10.310966	8.4169933	7.6020400	-0.0381	-2.80577	-2.223883333	-0.1436	-2.037573333	-2.852526667	3
10248	HSPA1A	heat shock	10.913766	13.629900	10.933966	11.046766	13.587433	11.039633	10.376300	2.716133333	0.0202	0.133	2.673666667	0.125866667	-0.537466667	4
10249	HSPA1A //,	heat shock	10.017706	13.477033	10.100883	10.150153	13.346533	10.107553	9.5208133	3.459326667	0.083176667	0.132446667	3.328826667	0.089846667	-0.496893333	4
12077	SERPINE1	serpin pep	10.869433	12.075933	12.286200	12.131533	12.802566	13.229466	13.207533	1.2065	1.416766667	1.2621	1.933133333	2.360033333	2.3381	4
16423	HSPH1	heat shock	9.7572133	12.251166	10.400499	10.655099	12.160299	10.750366	10.147533	2.493953333	0.643286667	0.897886667	2.403086667	0.993153333	0.39032	4
316	CARD16	caspase re	3.7235500	3.3428833	6.3420000	6.4535266	3.4320466	6.8131999	7.1116966	-0.380666667	2.61845	2.729976667	-0.291503333	3.08965	3.388146667	5
1499	C1orf187	chromosor	4.6556033	5.5085433	7.9330366	7.802223	5.6831866	7.6044033	7.5776333	0.85294	3.277433333	3.14662	1.027583333	2.9488	2.92203	5
4098	FN1	fibronectin	5.9133366	6.4640333	7.4140299	7.2574599	7.2663433	8.2901566	8.5927900	0.550696667	1.500693333	1.344123333	1.353006667	2.37682	2.679453333	5
10400	CCND2	cyclin D2	6.0097133	5.3066333	8.4271833	8.6134533	5.4533500	7.5484633	7.7392166	-0.70308	2.41747	2.60374	-0.556363333	1.53875	1.729503333	5
11292	EFEMP1	EGF-contai	5.3833766	6.3853933	6.9199866	7.6314700	6.9946633	9.5819833	9.0874233	1.002016667	1.53661	2.248093333	1.611286667	4.198606667	3.704046667	5
11431	PAPPA	pregnancy-	5.1076833	5.1230966	6.8276133	6.7425566	5.0021433	7.7301199	7.4435933	0.015413333	1.71993	1.634873333	-0.10554	2.622436667	2.33591	5
11524	PLTP	phospholip	5.6899500	5.1115033	8.0973833	7.6251433	5.4693900	8.0649700	8.2238666	-0.578446667	2.407433333	1.935193333	-0.22056	2.37502	2.533916667	5
11535	MX1	myxovirus	9.3982133	6.9618166	6.9147133	8.1580933	7.3093200	8.2670466	6.3168333	-2.436396667	-2.4835	-1.24012	-2.088893333	-1.131166667	-3.08138	5
12398	IL1R1	interleukin	5.8688866	5.4058233	5.7583800	5.9033233	7.0755666	8.7345633	8.5184666	-0.463063333	-0.110506667	0.034436667	1.20668	2.865676667	2.64958	5
12780	INPP5D	inositol pol	6.3934433	5.2931066	8.9124300	8.1773000	6.0598266	8.1244466	9.1749533	-1.100336667	2.518986667	1.783856667	-0.333616667	1.731003333	2.78151	5
13010	FEZ1	fasciculatic	5.3673166	4.8414966	7.6130233	7.4227633	5.3206366	7.7249133	7.8755799	-0.52582	2.245706667	2.055446667	-0.04668	2.357596667	2.508263333	5
13562	NID2	nidogen 2 (	6.0419200	6.2992433	6.4309166	6.5373966	6.1692366	8.6507766	8.8151400	0.257323333	0.388996667	0.495476667	0.127316667	2.608856667	2.77322	5
13622	ALOX5AP	arachidona	5.4081366	5.0135833	5.7073700	5.5978733	6.5082233	8.4228900	7.8691999	-0.394553333	0.299233333	0.189736667	1.100086667	3.014753333	2.461063333	5
13768	COL11A1	collagen, ty	4.8260900	4.6386100	6.9088233	6.0403566	4.5496200	7.4565800	8.1933600	-0.18748	2.082733333	1.214266667	-0.27647	2.63049	3.36727	5
14146	ISG20	interferon	8.8291233	8.0933700	6.1791600	7.5120800	7.6785833	6.8840300	5.8599366	-0.735753333	-2.649963333	-1.317043333	-1.15054	-1.945093333	-2.969186667	5
14278	PSG5	pregnancy	4.7702833	5.2112400	8.2254533	7.6689433	5.0825833	6.9265000	8.3753499	0.440956667	3.45517	2.89866	0.3123	2.156216667	3.605066667	5
14947	SRPX2	sushi-repea	6.2996333	6.5501900	8.0427533	7.9573466	6.6774566	8.8561666	8.6543566	0.250556667	1.74312	1.657713333	0.377823333	2.556533333	2.354723333	5
15108	OASL	2'-5'-oligoa	8.7344533	8.2890500	5.9914733	8.1160433	7.8423733	8.0194566	5.7808100	-0.445403333	-2.74298	-0.61841	-0.89208	-0.714996667	-2.953643333	5
15611	CLCA2	chloride ch	4.5812666	4.6055466	7.2463700	6.5201433	4.4653633	6.3450000	7.2395366	0.02428	2.665103333	1.938876667	-0.115903333	1.763733333	2.65827	5
15612	CLCA2	chloride ch	4.6970400	4.2393300	8.9390333	8.0321200	4.4677466	7.8884533	8.9293900	-0.45771	4.241993333	3.33508	-0.229293333	3.191413333	4.23235	5
15613	CLCA2	chloride ch	3.9949166	4.0818400	7.3270133	6.7885600	4.0603266	6.5390100	7.5614500	0.086923333	3.332096667	2.793643333	0.06541	2.544093333	3.566533333	5
15879	HAS2	hyaluronar	5.9589166	7.2761033	9.2217533	8.5332700	5.7049366	7.9259966	8.0676366	1.317186667	3.262836667	2.574353333	-0.25398	1.96708	2.10872	5
15887	LIN7A	lin-7 homo	4.3413700	4.9014033	5.8447333	6.0546966	5.1416366	6.8604099	6.7185733	0.560033333	1.503363333	1.713326667	0.800266667	2.51904	2.377203333	5
16078	PTGER2	prostaglan	5.2395800	5.8978866	5.1337433	5.0794133	7.2509733	8.2557133	8.3086533	0.658306667	-0.105836667	-0.160166667	2.011393333	3.016133333	3.069073333	5
18513	JAG1	jagged 1	4.0562966	4.8060833	5.5449666	5.3741933	4.7898299	6.4861133	7.9101633	0.749786667	1.48867	1.317896667	0.733533333	2.429816667	3.853866667	5
18806	ENPP2	ectonucleo	5.1925533	4.3854066	5.9913799	5.6394200	4.8617600	7.6411066	7.8827966	-0.807146667	0.798826667	0.446866667	-0.330793333	2.448553333	2.690243333	5
19102	ASTN2	astrotactin	5.4648600	6.2818499	8.0053199	8.2607733	6.1216033	8.1008266	7.5786833	0.81699	2.54046	2.795913333	0.656743333	2.635966667	2.113823333	5
19927	BIRC3	baculoviral	5.8020966	6.5852933	7.4934166	6.5985800	6.7689266	8.2861666	9.0207999	0.783196667	1.69132	0.796483333	0.96683	2.48407	3.218703333	5
20705	CASP1	caspase 1,	4.2686199	3.4301266	6.2505099	6.2125766	3.5115533	7.1243666	6.9452333	-0.838493333	1.98189	1.943956667	-0.757066667	2.855746667	2.676613333	5
21864	SPRY1	sprouty ho	4.9000933	4.8476133	5.6268933	5.7397766	5.6215333	7.5529766	8.3374966	-0.05248	0.7268	0.839683333	0.72144	2.652883333	3.437403333	5
23359	IFI44	Interferon-	8.1607900	7.4896966	5.7351166	7.1115300	7.3032866	7.0685800	5.4910666	-0.671093333	-2.425673333	-1.04926	-0.857503333	-1.09221	-2.669723333	5
24702	ASTN2	astrotactin	5.5735966	6.0976666	7.9570400	8.0590733	6.0928200	7.8438800	7.5118600	0.52407	2.383443333	2.485476667	0.519223333	2.270283333	1.938263333	5
26131	LAMA2	laminin, alp	5.1318966	4.7995600	5.5268500	5.0065300	5.1129566	7.5941433	9.4921233	-0.332336667	0.394953333	-0.125366667	-0.01894	2.462246667	4.360226667	5
26788	IFIT2	interferon-	8.5495300	8.6785300	5.8179633	8.0813766	8.1041499	7.5169033	5.7750466	0.129	-2.731566667	-0.468153333	-0.44538	-1.032626667	-2.774483333	5
26814	CLCA2	chloride ch	4.4281666	4.3199566	8.4319966	7.5149900	4.0785333	7.3720900	8.5247566	-0.10821	4.00383	3.086823333	-0.349633333	2.943923333	4.09659	5
26860	CDH8	cadherin 8,	4.4202966	5.3200933	6.8741300	7.0176500	5.0902833	7.1976933	7.3343166	0.899796667	2.453833333	2.597353333	0.669986667	2.777396667	2.91402	5
29301	AHNAK	AHNAK nuc	8.8372066	8.5739833	6.0617733	6.5780033	8.5346433	6.7151100	6.2180333	-0.263223333	-2.775433333	-2.259203333	-0.302563333	-2.122096667	-2.619173333	5
29400	CDH10	cadherin 1(	4.0726266	4.3933433	7.0922133	6.7368066	4.5172766	6.5739166	6.9825300	0.320716667	3.019586667	2.66418	0.44465	2.50129	2.909903333	5
30071	SLC38A4	solute carr	6.0309500	6.6268533	7.3749766	7.5989166	7.5403966	8.8650766	8.7502400	0.595903333	1.344026667	1.567966667	1.509446667	2.834126667	2.71929	5
31122	KLF4	Kruppel-lik	8.4069666	7.1316766	5.7670600	7.6551033	6.7881300	7.4652266	6.0216266	-1.27529	-2.639906667	-0.751863333	-1.618836667	-0.94174	-2.38534	5
35958	CMPK2	cytidine m	8.2575000	6.6407433	5.0734233	7.2817066	6.3756800	6.9016100	4.3485366	-1.616756667	-3.184076667	-0.975793333	-1.88182	-1.35589	-3.908963333	5
37184	---	---	5.6469733	6.1713866	7.7301133	7.6688333	6.4957233	8.6098066	9.1587766	0.524413333	2.08314	2.02186	0.84875	2.962833333	3.511803333	5
37203	FGD4	FYVE, RhoC	5.2406600	4.8554299	5.9843766	5.2161266	6.4218233	7.7520566	9.0503300	-0.38523	0.743716667	-0.024533333	1.181163333	2.511396667	3.80967	5
38586	SPATA18	spermatog	4.6913500	4.5050400	7.1827833	6.6267300	4.7226466	7.4131900	8.7159033	-0.18631	2.491433333	1.93538	0.031296667	2.72184	4.024553333	5
40434	IP6K3	inositol he:	5.6805100	5.1055066	7.4817666	6.9648966	6.6125100	8.6136166	8.9085699	-0.575003333	1.801256667	1.284386667	0.932	2.933106667	3.22806	5

40438	JAG1	Jagged 1 (A	5.1161433	6.3373200	7.1620533	6.9195466	6.2784300	7.8209766	8.9387133	1.221176667	2.04591	1.803403333	1.162286667	2.704833333	3.82257	5
40733	PDE4C	phosphodi	3.4612066	3.6545433	6.6613766	6.3987900	4.0166233	7.6904233	8.3638566	0.193336667	3.20017	2.937583333	0.555416667	4.229216667	4.90265	5
41030	TNFRSF10A	tumor necr	5.7961533	5.9824966	7.5826933	7.2681766	6.3247066	8.1531633	8.5720900	0.186343333	1.78654	1.472023333	0.528553333	2.35701	2.775936667	5
42002	PAPPA	pregnancy-	3.9167533	4.0827566	5.8685566	5.2428300	4.4617700	6.4094833	7.4087733	0.166003333	1.951803333	1.326076667	0.545016667	2.49273	3.49202	5
42807	SULF2	sulfatase 2	4.8764066	4.7336133	7.4975533	7.2630333	5.0014666	7.6628266	7.7644566	-0.142793333	2.621146667	2.386626667	0.12506	2.78642	2.88805	5
44911	---	---	5.7006700	5.7331933	8.2282666	7.5756233	5.1648966	7.0455233	8.0620166	0.032523333	2.527596667	1.874953333	-0.535773333	1.344853333	2.361346667	5
46456	MYOCD	myocardin	4.5239166	6.6812700	7.8670900	7.2650633	5.7769066	6.8730000	7.0027366	2.157353333	3.343173333	2.741146667	1.25299	2.349083333	2.47882	5
49478	CSMD3	CUB and Su	4.6779100	4.7342200	7.6142433	6.7947500	4.5790500	6.3417900	7.5956166	0.05631	2.936333333	2.11684	-0.09886	1.66388	2.917706667	5
49494	---	---	7.3703866	4.5953399	7.0192666	7.2661933	5.0178000	6.8773599	6.8596266	-2.775046667	-0.35112	-0.104193333	-2.352586667	-0.493026667	-0.51076	5
49700	---	---	4.0478066	4.9569166	7.4660100	6.8751133	5.3384433	8.3604299	8.8506966	0.90911	3.418203333	2.827306667	1.290636667	4.312623333	4.80289	5
52337	WDR63	WD repeat	4.0056633	4.3365866	6.6398100	6.6774133	4.3529400	6.8626366	6.8874266	0.330923333	2.634146667	2.67175	0.347276667	2.856973333	2.881763333	5
54181	ISG20	interferon	8.6837433	7.8296500	6.0065866	7.1865100	7.5012400	6.7145933	5.6445266	-0.854093333	-2.677156667	-1.497233333	-1.182503333	-1.96915	-3.039216667	5
54295	COL11A1	collagen, ty	4.4607766	4.3241166	7.0951633	5.9864900	4.2567333	7.6612566	8.4583600	-0.13666	2.634386667	1.525713333	-0.204043333	3.20048	3.997583333	5
1902	ABCD3	ATP-bindin	6.9419200	7.0768933	4.2969500	4.6005433	6.0186566	4.7257600	4.3012433	0.134973333	-2.64497	-2.341376667	-0.923263333	-2.21616	-2.640676667	6
4493	LOC10012	hypothetic	3.3737900	3.3241866	5.7093900	4.2517333	3.1773800	4.2580400	5.8535333	-0.049603333	2.3356	0.877943333	-0.19641	0.88425	2.479743333	6
10401	CCND2	cyclin D2	4.2809533	3.8603433	6.6247866	6.7490433	4.4266700	5.5246966	5.8863433	-0.42061	2.343833333	2.46809	0.145716667	1.243743333	1.60539	6
11825	SERPINA3	serpin pep	4.6655433	4.3592200	4.6896433	4.6166433	4.9424066	7.3180200	7.0602033	-0.306323333	0.0241	-0.0489	0.276863333	2.652476667	2.39466	6
15459	CASP1	caspase 1,	3.6083266	3.4074000	5.8398466	5.1953933	3.4870400	6.1573733	6.5849033	-0.200926667	2.23152	1.587066667	-0.121286667	2.549046667	2.976576667	6
16034	CCT6B	chaperonir	5.8899366	4.4346233	3.6076866	3.5556766	4.5440733	3.6363200	3.5191966	-1.455313333	-2.28225	-2.33426	-1.345863333	-2.253616667	-2.37074	6
19975	INHBE	inhibin, be	6.2179766	3.7641000	4.4657933	4.7804800	3.7040000	4.6776700	3.8657800	-2.453876667	-1.752183333	-1.437496667	-2.513976667	-1.540306667	-2.352196667	6
29074	NPR3	natriuretic	4.5493333	5.3328866	4.6095266	5.0176600	5.5802233	7.4944766	6.9255600	0.783553333	0.060193333	0.468326667	1.03089	2.945143333	2.376226667	6
31589	LOC10050	hypothetic	6.6934133	5.5781200	4.2690600	4.3869300	5.4342766	4.7206700	4.0896000	-1.115293333	-2.424353333	-2.306483333	-1.259136667	-1.972743333	-2.603813333	6
32865	THAP2	THAP dom	4.1674466	6.7447166	5.2230466	5.7121966	6.6898366	5.6075300	5.3798100	2.57727	1.0556	1.54475	2.52239	1.440083333	1.212363333	6
38109	---	---	2.9304466	2.9834033	2.8966000	2.9687066	3.0027033	6.2794466	7.4788499	0.052956667	-0.033846667	0.03826	0.072256667	3.349	4.548403333	6
40759	CCDC148	coiled-coil	4.2844833	4.2678800	7.0957566	6.6606800	3.9093466	4.3906633	4.5826033	-0.016603333	2.811273333	2.376196667	-0.375136667	0.10618	0.29812	6
44294	CYYR1	cysteine/ty	3.3003566	3.5154633	5.3824900	5.5296566	3.6530033	5.8286100	5.7180100	0.215106667	2.082133333	2.2293	0.352646667	2.528253333	2.417653333	6
48169	---	---	3.5336566	4.1228100	4.0135266	4.2537166	5.2423533	6.1152500	6.6553066	0.589153333	0.47987	0.72006	1.708696667	2.581593333	3.12165	6
48900	NCKAP5	NCK-associ	3.1536766	3.3425566	5.7638466	5.5528100	2.9320000	5.4073299	6.7246633	0.18888	2.61017	2.399133333	-0.221676667	2.253653333	3.570986667	6
