# Supplementary material for: Genome-Wide Chromatin Landscape Transitions Identify Novel Pathways in Early Commitment to Osteoblast Differentiation
Source: PLoS One. 2016 Feb 18;11(2):e0148619. doi: 10.1371/journal.pone.0148619 (PMC4759368; doi:10.1371/journal.pone.0148619)
Supplement: S5 Table — This group of genes has a p value of 6. 5x10^-19^. Annotation for each gene was performed as described in Methods. (PDF) [file pone.0148619.s016.pdf]

Symbol	Synonym(	Entrez Ge	Entrez Ge	Intensity_	Delta_Log	Location	Family
**abnormal morphology of bone**							function
**ADAMTS2**	a disintegr	ADAM me	NM_0215	15.0	1.1254766	Extracellul	peptidase
**ALKBH1**	2700073G	alkB, alkylation repair homolog 1			0.5684066	Cytoplasm	enzyme
**ALPL**	AKP2, ALI	alkaline ph	NM_0011	19.7	0.8530433	Plasma Me	phosphatase
**ALX4**	ALX home	ALX home	NM_0219	16.3	-0.088056	Nucleus	transcription regulator
**AMER1**	28100020	APC mem	NM_1524	11.9	-0.072413	Plasma Me	other
**APOE**	AD2, AI25	apolipopro	NM_0000	16.6	0.5746566	Extracellul	transporter
**BMP4**	BMP2B, B	bone morp	NM_1308	16.3	0.1652333	Extracellul	growth factor
**BMP7**	bone morp	bone morp	NM_0017	11.3	-0.086506	Extracellul	growth factor
**CCND1**	AI327039,	cyclin D1	NM_0530	26.3	0.0574033	Nucleus	transcription regulator
**CDKN1A**	CAP20, CI	cyclin-dep	NM_0003	14.4	1.7663000	Nucleus	kinase
**CHSY1**	CHONDR	chondroitir	NM_0149	27.9	-0.067766	Cytoplasm	enzyme
**COL11A2**	collagen, t	collagen, type XI, alpha 2			-0.182693	Extracellul	other
**COL12A1**	AW74388	collagen, t	NM_0043	5.3	0.8381266	Extracellul	other
**COL18A1**	COLLAGE	collagen, t	NM_1304	50.4	-0.118236	Extracellul	other
**COL2A1**	ANFH, A	collagen, t	NM_0018	1.3	0.2310433	Extracellul	other
**CREBBP**	AW55829	CREB binc	NM_0043	21.6	-0.345526	Nucleus	transcription regulator
**CYP27B1**	1-Hydroxyl	cytochrome P450, family 27, su			-0.138749	Cytoplasm	enzyme
**DLL3**	delta-like 3	delta-like 3 (Drosophila)			-0.483429	Extracellul	other
**DLX1**	distal-less	distal-less homeobox 1			-0.246726	Nucleus	transcription regulator
**DMRT2**	doublesex	doublesex	NM_0065	15.0	-0.135549	Nucleus	other
**DVL1**	DISHEVEI	dishevelled segment polarity pro			-0.026080	Cytoplasm	other
**DVL3**	dishevelle	dishevelled segment polarity pro			0.2992433	Cytoplasm	other
**E2F3**	E2F trans	E2F trans	NM_0019	12.5	0.0192633	Nucleus	transcription regulator
**EFNB1**	Cek5-L, CI	ephrin-B1	NM_0044	13.8	-0.024863	Plasma Me	other
**EGLN2**	0610011A	egl-9 family hypoxia-inducible fa			-0.157196	Cytoplasm	enzyme
**ELN**	AI385707,	elastin	NM_0010	7.8	-0.071663	Extracellul	other
**EMX2**	empty spir	empty spir	NM_0011	13.5	0.0385899	Nucleus	transcription regulator
**EN1**	engrailed	engrailed	NM_0014	16.9	0.0662233	Nucleus	transcription regulator
**EPHB3**	AW45689	EPH recep	NM_0044	16.9	-0.35534	Plasma Me	kinase
**EYA1**	BOP, BOF	EYA trans	NM_0005	18.8	0.6492266	Nucleus	phosphatase
**FAM20C**	BC004044	family with	NM_0202	11.3	-0.64276	Extracellul	enzyme
**FBN1**	ACMICD,	fibrillin 1	NM_0001	14.1	2.3645200	Extracellul	other
**FGF3**	fibroblast g	fibroblast g	NM_0052	12.8	-0.056163	Extracellul	growth factor
**FGFR3**	ACH, CD3	fibroblast g	NM_0011	32.6	-0.75474	Plasma Me	kinase
**FGFRL1**	FGFR5, F	fibroblast g	NM_0219	21.6	-0.273646	Plasma Me	transmembrane receptor
**FKBP8**	FK506 bin	FK506 binding protein 8, 38kDa			-0.262149	Cytoplasm	other
**FOSL2**	FLJ23306,	FOS-like a	NM_0052	13.8	1.2218366	Nucleus	transcription regulator
**FOXF2**	Fkh20, FK	forkhead	NM_0014	14.7	-0.053370	Nucleus	transcription regulator
**FOXI3**	forkhead	forkhead	NM_0011	3.4		Nucleus	transcription regulator
**FST**	AL033346	follistatin	NM_0134	7.5	0.3158900	Extracellul	other
**FZD9**	CD349, fri	frizzled cla	NM_0035	23.8	-0.308193	Plasma Me	G-protein coupled receptor
**GAD1**	CPSQ1, E	glutamate	NM_0134	13.5	-0.161750	Cytoplasm	enzyme
**GBX2**	D130058E	gastrulatio	NM_0014	26.0	-0.104473	Nucleus	transcription regulator
**GDF6**	BMP-13, C	growth diff	NM_0010	18.5		Extracellul	growth factor
**GLI2**	AW54612	GLI family	NM_0052	19.4	-0.203066	Nucleus	transcription regulator
**GNA11**	BOS 7750	guanine nu	NM_0020	19.7	0.1356299	Plasma Me	enzyme
**GNAI3**	87U6, AI1	guanine nucleotide binding prote			-0.331566	Cytoplasm	enzyme
**GPER1**	6330420K	G protein-	NM_0010	16.9	-0.610016	Plasma Me	G-protein coupled receptor
**GPR55**	G protein-	G protein-coupled receptor 55			-0.120876	Plasma Me	G-protein coupled receptor
**H19**	AI747191,	H19, impri	NR_0021	15.4	1.2130100	Cytoplasm	other
**HIVEP3**	2900056N	human imr	NM_0245	17.9	1.9700033	Nucleus	transcription regulator
**HMX3**	H6 family	H6 family	NM_0011	7.5		Nucleus	transcription regulator
**HOXA1**	BSAS, ER	homeobox A1			0.0735833	Nucleus	transcription regulator

**HOXA2**	AI324701,	homeobox	NM_0067	19.7	-0.131299	Nucleus	transcription regulator
**HOXA5**	homeo bo	homeobox	NM_0191	18.2	-0.157173	Nucleus	transcription regulator
**HOXA9**	ABD-B, D	homeobox A9				Nucleus	transcription regulator
**HOXB2**	AI894218,	homeobox	NM_0021	31.6	-0.194786	Nucleus	transcription regulator
**HOXB3**	homeo bo	homeobox	NM_0021	9.4	-0.142913	Nucleus	transcription regulator
**HOXB4**	B4, home	homeobox B4			0.1056433	Nucleus	transcription regulator
**HOXB9**	homeo bo	homeobox	NM_0240	21.6	-0.700233	Nucleus	transcription regulator
**HOXC4**	cp19, hom	homeobox	NM_1536	19.4	-0.038053	Nucleus	transcription regulator
**HOXC6**	CP25, HH	homeobox C6			0.1532266	Nucleus	transcription regulator
**HOXD10**	AI385591,	homeobox D10			-0.048183	Nucleus	transcription regulator
**HOXD11**	E230017H	homeobox D11			-0.242786	Nucleus	transcription regulator
**HOXD12**	homeo bo	homeobox D12			-0.067069	Nucleus	transcription regulator
**HOXD3**	homeo bo	homeobox	NM_0068	32.9	0.0895633	Nucleus	transcription regulator
**HOXD4**	6030436D	homeobox D4			0.1531766	Nucleus	transcription regulator
**HS6ST1**	6Ost1, he	heparan s	NM_0048	17.5	0.506190	Plasma M	enzyme
**HSPG2**	AI852380,	heparan s	NM_0055	19.7	0.9481366	Extracellul	enzyme
**IGFALS**	ACLSD, A	insulin-like	NM_0011	21.0	-0.304610	Extracellul	other
**INPP5E**	1200002L	inositol pol	NM_0198	16.3	-0.080086	Cytoplasm	phosphatase
**ITGA3**	AA407068	integrin, al	NM_0022	18.2	0.1801666	Plasma M	other
**ITGB2**	2E6, AI52	integrin, b	NM_0011	18.8	-0.268663	Plasma M	transmembrane receptor
**JAG2**	D12Ggc2	jagged 2	NM_1451	17.9	0.2614733	Extracellul	growth factor
**KCNQ1**	ATFB1, A	potassium	NM_1817	17.5	-0.146573	Plasma M	ion channel
**KIF7**	ACLS, HL	kinesin fan	NM_1985	12.2	-0.163450	Extracellul	other
**KLF10**	AI115143,	Kruppel-lik	NM_0056	8.5	-0.177640	Nucleus	transcription regulator
**KLF2**	Kruppel-lik	Kruppel-lik	NM_0162	21.0	-1.2508	Nucleus	transcription regulator
**LFNG**	AW06116	LFNG O-f	NM_0010	23.5	-0.183633	Cytoplasm	enzyme
**LIF**	CDF, choli	leukemia i	NM_0023	10.7	0.3438566	Extracellul	cytokine
**LMX1B**	LIM home	LIM home	NM_0011	17.5	-0.173016	Nucleus	transcription regulator
**LRP5**	BMND1, E	low density	NM_0023	62.3	0.3459566	Plasma M	transmembrane receptor
**LRRK1**	AW31959	leucine-ric	NM_0246	16.3	0.3609866	Cytoplasm	kinase
**LTBP3**	Latent tra	latent tran	NM_0011	18.8	0.5269566	Extracellul	other
**MATN1**	CMP, CR	matrilin 1, cartilage matrix protei			-0.178276	Extracellul	other
**MDFI**	I-MF, I-mf	MyoD fami	NM_0055	12.2	-0.133676	Cytoplasm	other
**MEOX1**	AI385561,	mesenchy	NM_0045	27.3	-0.310556	Nucleus	transcription regulator
**MFAP2**	AI893631,	microfibril	NM_0011	17.2	0.017060	Extracellul	other
**MFNG**	AL591946	MFNG O-f	NM_0024	14.4	0.1817366	Cytoplasm	enzyme
**MNT**	bHLHd3, M	MAX netw	NM_0203	17.5	-0.238086	Nucleus	transcription regulator
**MSI1**	m-Msi-1, M	musashi R	NM_0024	56.1	-0.204989	Cytoplasm	other
**MYF5**	B130010J	myogenic factor 5			2.3756099	Nucleus	transcription regulator
**MYOG**	bHLHc3, M	myogenin (myogenic factor 4)			0.674790	Nucleus	transcription regulator
**NAB2**	AI451907,	NGFI-A binding protein 2 (EGR1			-0.220293	Nucleus	transcription regulator
**NACC1**	2010001H	nucleus accumbens associated			-0.369146	Nucleus	transcription regulator
**NAGLU**	alpha-N-a	N-acetylglucosaminidase, alpha			-0.258659	Cytoplasm	enzyme
**NFIC**	1110019L	nuclear fa	NM_0055	17.9	0.1972633	Nucleus	transcription regulator
**NFIX**	CTF, MRS	nuclear fa	NM_0025	26.0	-0.629200	Nucleus	transcription regulator
**NOS3**	2310065A	nitric oxide	NM_0011	14.4	-0.335076	Cytoplasm	enzyme
**NPR2**	AMDM, Ar	natriuretic peptide receptor 2			0.808210	Plasma M	G-protein coupled receptor
**OTX1**	A730044F	orthodenticle homeobox 1			-0.098090	Nucleus	transcription regulator
**PAX1**	hbs, hunc	paired box	NM_0061	17.9	0.1059333	Nucleus	transcription regulator
**PAX2**	FSGS7, O	paired box	NM_0002	21.0	0.0403599	Nucleus	transcription regulator
**PAX7**	HUP1, LO	paired box	NM_0011	14.7	0.2874033	Nucleus	transcription regulator
**PAX8**	paired box	paired box	NM_0139	20.7	-0.223699	Nucleus	transcription regulator
**PITX1**	BFT, CCF	paired-like	NM_0026	13.5	-0.174549	Nucleus	transcription regulator
**PITX2**	9430085M	paired-like	NM_0003	22.6	0.4241599	Nucleus	transcription regulator
**PKD1**	mFLJ002	polycystic	NM_0002	43.9	-0.238703	Plasma M	ion channel
**PLXNA1**	2600013D	plexin A1	NM_0322	13.8	0.472350	Plasma M	transmembrane receptor

**PLXND1**	6230425C	plexin D1	NM_0151(	11.3	0.5124633	Plasma Me	transmembrane receptor
**POU3F3**	Brain1, BF	POU class	NM_0062:	14.7	0.0519533	Nucleus	transcription regulator
**PTCH1**	A230106A	patched 1	NM_0010{	15.0	-0.494986(	Plasma Me	transmembrane receptor
**PTHLH**	BDE2, HH	parathyroi(	NM_1989(	8.5	-0.626643:	Extracellul	other
**PTPN2**	AI325124,	protein tyr(	NM_0028:	21.3	-0.428996(	Cytoplasm	phosphatase
**RAI1**	DOM3, Gt	retinoic ac	NM_0306(	13.5	-0.384676(	Cytoplasm	other
**RFNG**	RFNG O-fı	RFNG O-fucosylpeptide 3-beta-N			0.4347433	Cytoplasm	enzyme
**RGMA**	BC059072	repulsive ç	NM_0202'	18.2	0.1420733	Plasma Me	other
**RING1**	DADB-10C	ring finger	NM_0029:	25.7	-0.135586(	Nucleus	transcription regulator
**ROR2**	BDB, BDB	receptor ty	NM_0045(	16.9	-0.220513:	Plasma Me	kinase
**SBNO2**	BC01920(	strawberry notch homolog 2 (Drc			-0.096030(	Other	transcription regulator
**SHH**	95300360	sonic hedç	NM_0001(	13.2	-0.137416(	Extracellul	peptidase
**SIX1**	BB138287	SIX homeobox 1			0.1306999	Nucleus	transcription regulator
**SKI**	2310012I(	SKI proto-(	NM_0030:	31.6	0.449190(	Nucleus	transcription regulator
**SLC39A1**	BC02153(	solute carr	NM_0011:	12.5	0.14337	Plasma Me	transporter
**SLC4A2**	AE2, Aep2	solute carrier family 4 (anion exc			-0.208226(	Plasma Me	transporter
**SMAD3**	AU022421	SMAD fan	NM_0059(	15.0	0.2322199	Nucleus	transcription regulator
**SOX9**	2010306G	SRY (sex (	NM_000З4	8.1	-1.052923:	Nucleus	transcription regulator
**SRC**	ASV, AW2	SRC prot(	NM_1982(	13.5	0.194060(	Cytoplasm	kinase
**TBX1**	CAFS, CA	T-box 1	NM_0059(	18.2	-0.330773:	Nucleus	transcription regulator
**TBX5**	HOS, T-bc	T-box 5	NM_0001(	16.6	-0.201773:	Nucleus	transcription regulator
**TCF15**	BHLH-EC:	transcription factor 15 (basic heli			-0.222209(	Nucleus	transcription regulator
**TCIRG1**	a3, ATP6a	T-cell, imn	NM_0060!	15.4	1.0072999	Plasma Me	enzyme
**TFAP2A**	activating (	transcription factor AP-2 alpha (a			-0.126843:	Nucleus	transcription regulator
**THRA**	6430529J(	thyroid hormone receptor, alpha			0.4276233	Nucleus	ligand-dependent nuclear receptor
**TMEM119**	AW20894(	transmeml	NM_1817:	12.5	0.9146966	Cytoplasm	other
**TP73**	P73, p73R	tumor prot(	NM_0011:	20.4	-0.082410(	Nucleus	transcription regulator
**TSHZ1**	5730407I(	teashirt zir	NM_0057(	14.1	-0.609563:	Nucleus	transcription regulator
**UNCX**	Chx4, LO(	UNC hom(	NM_0010(	36.7	0.1420733	Other	transcription regulator
**VDR**	BOS 5154	vitamin D (1,25- dihydroxyvitami			0.2452666	Nucleus	transcription regulator
**VEGFA**	BOS 2146	vascular e	NM_0010:	3.1	0.3762433	Extracellul	growth factor
**WFIKKN1**	C16orf12,	WAP, follistatin/kazal, immunogl(			-0.091139(	Cytoplasm	other
**WNT1**	BMND16,	wingless-type MMTV integration			-0.113279(	Extracellul	cytokine
**WNT3A**	RP23-378I	wingless-t	NM_0331:	13.2	0.14337	Extracellul	cytokine
**WNT5A**	8030457G	wingless-t	NM_0033(	16.6	1.7833199	Extracellul	cytokine
**WNT9A**	RP23-378I	wingless-t	NM_0033(	15.7	-0.200350(	Extracellul	other
**ZBTB16**	AI467657,	zinc finger	NM_0010'	12.2	1.5429566	Nucleus	transcription regulator
**ZIC5**	1700049L:	Zic family (	NM_0331:	12.5	0.0435033	Nucleus	transcription regulator
**ZNF521**	B930086A	zinc finger	NM_0154(	6.9	0.1438333	Nucleus	other
