# Supplementary material for: Genome-Wide Chromatin Landscape Transitions Identify Novel Pathways in Early Commitment to Osteoblast Differentiation
Source: PLoS One. 2016 Feb 18;11(2):e0148619. doi: 10.1371/journal.pone.0148619 (PMC4759368; doi:10.1371/journal.pone.0148619)
Supplement: S6 Table — (PDF) [file pone.0148619.s017.pdf]

S6 Table

http://www.ncbi.nlm.nih.gov/geo/query/acc.cgi?acc=GSE75232.

DHS	Sample name	characteristics: treatment
1	B34 rep#1 DNase-seq	untreated, grow at 34oC in BM
2	B34 rep#2 DNase-seq	untreated, grow at 34oC in BM
3	B39-d1 rep#1 DNase-seq	untreated, grow at 39oC in BM
4	B39-d1 rep#2 DNase-seq	untreated, grow at 39oC in BM
5	B39-d2 rep#1 DNase-seq	untreated, grow at 39oC in BM
6	B39-d2 rep#2 DNase-seq	untreated, grow at 39oC in BM
7	OIM-d1 rep#1 DNase-seq	grow at 39oC in OIM
8	OIM-d1 rep#2 DNase-seq	grow at 39oC in OIM
9	OIM-d2 rep#1 DNase-seq	grow at 39oC in OIM
10	Oim-d2 rep#2 DNase-seq	grow at 39oC in OIM
**Micriarray**	**Sample name**	**characteristics: treatment**
1	B34BM_1	untreated, grow at 34oC in BM
2	B34BM_2	untreated, grow at 34oC in BM
3	B34BM_3	untreated, grow at 34oC in BM
4	B39_4hr_1	untreated, grow at 34oC in BM
5	B39_4hr_2	untreated, grow at 39oC in BM
6	B39_4hr_3	untreated, grow at 39oC in BM
7	B39_24hr_1	untreated, grow at 39oC in BM
8	B39_24hr_2	untreated, grow at 39oC in BM
9	B39_24hr_3	untreated, grow at 39oC in BM
10	B39_48hr_1	untreated, grow at 39oC in BM
11	B39_48hr_2	untreated, grow at 39oC in BM
12	B39_48hr_3	untreated, grow at 39oC in BM
13	OIM_4hr_1	OIM treated, grow at 39oC
14	OIM_4hr_2	OIM treated, grow at 39oC
15	OIM_4hr_3	OIM treated, grow at 39oC
16	OIM_24hr_1	OIM treated, grow at 39oC
17	OIM_24hr_2	OIM treated, grow at 39oC
18	OIM_24hr_3	OIM treated, grow at 39oC
19	OIM_48hr_1	OIM treated, grow at 39oC
20	OIM_48hr_2	OIM treated, grow at 39oC
21	OIM_48hr_3	OIM treated, grow at 39oC
